# A scoping review of distributed ledger technology in genomics: thematic analysis and directions for future research

**DOI:** 10.1093/jamia/ocac077

**Published:** 2022-05-20

**Authors:** Mikael Beyene, Philipp A Toussaint, Scott Thiebes, Matthias Schlesner, Benedikt Brors, Ali Sunyaev

**Affiliations:** Department of Economics and Management, Karlsruhe Institute of Technology, Karlsruhe, Germany; HIDSS4Health—Helmholtz Information and Data Science School for Health, Karlsruhe/Heidelberg, Germany; Department of Economics and Management, Karlsruhe Institute of Technology, Karlsruhe, Germany; HIDSS4Health—Helmholtz Information and Data Science School for Health, Karlsruhe/Heidelberg, Germany; Department of Economics and Management, Karlsruhe Institute of Technology, Karlsruhe, Germany; Biomedical Informatics, Data Mining and Data Analytics, Faculty of Applied Computer Science and Medical Faculty, University of Augsburg, Augsburg, Germany; Bioinformatics and Omics Data Analytics, German Cancer Research Center (DKFZ), Heidelberg, Germany; Division of Applied Bioinformatics, German Cancer Research Center (DKFZ), Heidelberg, Germany; Translational Oncology, National Center for Tumor Diseases, German Cancer Research Center (DKFZ), Heidelberg, Germany; Department of Economics and Management, Karlsruhe Institute of Technology, Karlsruhe, Germany

**Keywords:** distributed ledger technology, Blockchain, genomics, literature review, thematic analysis

## Abstract

**Objective:**

Rising interests in distributed ledger technology (DLT) and genomics have sparked various interdisciplinary research streams with a proliferating number of scattered publications investigating the application of DLT in genomics. This review aims to uncover the current state of research on DLT in genomics, in terms of focal research themes and directions for future research.

**Materials and Methods:**

We conducted a scoping review and thematic analysis. To identify the 60 relevant papers, we queried Scopus, Web of Science, PubMed, ACM Digital Library, IEEE Xplore, arXiv, and BiorXiv.

**Results:**

Our analysis resulted in 7 focal themes on DLT in genomics discussed in literature, namely: (1) Data economy and sharing; (2) Data management; (3) Data protection; (4) Data storage; (5) Decentralized data analysis; (6) Proof of useful work; and (7) Ethical, legal, and social implications.

**Discussion:**

Based on the identified themes, we present 7 future research directions: (1) Investigate opportunities for the application of DLT concepts other than Blockchain; (2) Explore people’s attitudes and behaviors regarding the commodification of genetic data through DLT-based genetic data markets; (3) Examine opportunities for joint consent management via DLT; (4) Investigate and evaluate data storage models appropriate for DLT; (5) Research the regulation-compliant use of DLT in healthcare information systems; (6) Investigate alternative consensus mechanisms based on Proof of Useful Work; and (7) Explore DLT-enabled approaches for the protection of genetic data ensuring user privacy.

**Conclusion:**

While research on DLT in genomics is currently growing, there are many unresolved problems. This literature review outlines extant research and provides future directions for researchers and practitioners.

## INTRODUCTION

Distributed ledger technology (DLT) is a novel technology (eg, Blockchain) that facilitates the maintenance of digital, decentralized ledgers in networks of untrusted parties.^1^ Through coordinating procedures, called consensus mechanisms, all network participants maintain a single, uniform ledger across devices. This collectively maintained ledger then serves as ground truth for data used in applications built on top of it. Emerging in late 2008, the Bitcoin Blockchain was the first implementation of DLT. It provides a means for decentralized payments without relying on intermediaries (ie, financial institutions).^2^ Following this first generation of DLT, the Ethereum Blockchain went live in 2015. It represents the second generation of DLT and supports so-called smart contracts: decentralized applications running on the Ethereum network allowing for a myriad of use cases.

Due to the democratic philosophy inherent to DLT, embodying values such as equality and freedom, this consensus-driven technology has since left its home realm of financial transactions. It is now being applied in various other areas that deal with sensitive data, actors with misaligned incentives, or a general lack of trust. Besides uncommon and very recent application areas like digital art trade,^3^ DLT is especially applied in more conventional fields like supply-chain management, energy, real estate, and healthcare.^4–7^ Since an in-depth discussion of DLT is out of the scope of this paper, we provide an overview on DLT and related terminology for readers unfamiliar with DLT in Supplementary Material S1.

While the move into healthcare started with electronic health records, recently—also driven through the high availability of genomic data^8^—the number of applications of DLT in genomics has increased rapidly. This can be seen in particular in the prominent application in genetic data markets (eg, Nebula Genomics, EncrypGen, or LunaDNA). This is not a mere coincidence: Due to the transparency and security guarantees offered by DLT,^1^ the technology lends itself to handling people’s genes and genomes as their most personal and sensitive data. Whereas certain DLT attributes such as data integrity (ie, tamper resistance) are most recognized and, accordingly, are the focus of applications in genomics,^9^^,^^10^ there are other aspects of DLT that may lead to several novel applications. These range from DLT-based payment to changing the intrinsics of DLT to leverage the computational power of DLT networks for research analysis (ie, Proof of Useful Work).^11^^,^^12^

There are previous reviews covering some aspects of DLT in genomics (cf. Supplementary Table S2). Some address applications of DLT in genomics,^13^^,^^14^ others also address opportunities and challenges. The majority of reviews discuss the topic of DLT in genomics only marginally in the broader context of healthcare or the life sciences. Recently, a few reviews have also directly addressed the application of DLT in genomics,^10^^,^^15^^,^^16^ which indicates a growing research interest in the topic. Yet there is a lack of a comprehensive overview or a discussion of future research directions. Also, mainly due to the interdisciplinary nature of this research stream, existing reviews do not always reflect an in-depth understanding of DLT.

### Objectives

Given that the intersection of DLT and genomics has sparked new interdisciplinary research streams with a proliferating number of scattered publications, the present review article aims to answer the following research questions (RQ):

*RQ1—* *What is the current state of research on DLT in genomics in terms of focal research themes?*

*RQ2—* *How can these focal research themes inform future research on the topic?*

To summarize the recent developments on DLT in genomics and therefore answer RQ1, we present a scoping review of the literature and identify prevailing themes within.^17^ Subsequently, we discuss our results and provide directions for future research, thus answering RQ2.

## MATERIALS AND METHODS

**Figure 1.**
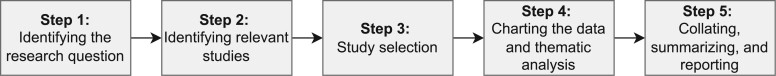
Overview of the literature review process adapted from Arksey and O’Malley.^18^

For the scoping review, we followed the framework of Arksey and O’Malley^18^ and augmented the data charting process with a thematic analysis after Braun and Clarke.^19^ Our review process unfolded as depicted in Figure 1. We also used the PRISMA scoping review checklist to guide the reporting of this review (see Supplementary Table S3).

### Identifying relevant studies

We queried 8 scientific databases for research articles and preprints with a broad search string (see Table 1). Due to the interdisciplinary nature of our research, we selected databases that index a wide array of scientific fields, including computer science, life and medical sciences, and information systems.

**Table 1.** Overview of the search strategy

Search string	(*“distributed ledger”* OR *“blockchain”* OR *“block chain”*) AND (*“genome”* OR *“genomes”* OR *“genomic”* OR *“genomics”* OR *“gene”* OR *“genes”* OR *“genetic”* OR *“genetics”* OR *“DNA”*)
Fields	Title; abstract; keywords
Databases	Regular databases: Scopus; Web of Science; PubMed; ACM Digital Library; IEEE Xplore Preprint databases: arXiv; BioRxiv
Publication types	Journal articles; conference papers; preprints
Date range	Peer-reviewed publications: January 2009 to August 2021 Preprints: January 2018 to August 2021
Additional literature	Forward and backward search on included literature

Since Bitcoin, as the first application of Blockchain or, more general, DLT, appeared in late 2008,^2^ we excluded research articles prior to 2009. We only included full papers published in peer-reviewed journals and conference proceedings written in English with the explicit exception of including preprints (from dedicated preprint databases). However, we also excluded preprints deposited prior to 01/2018, since most of these manuscripts are highly topical and older preprints are most likely already published. The initial search was conducted in 09/2020 and was updated in 08/2021; overall, resulting in 468 articles. Finally, we identified additional sources through a forward and backward search (*n* = 32) on our base literature set,^20^ and also manually added one more article to the list of potentially relevant studies (ie, Lemieux et al^21^) In total, we identified 501 potentially relevant studies.

### Study selection

**Figure 2.**
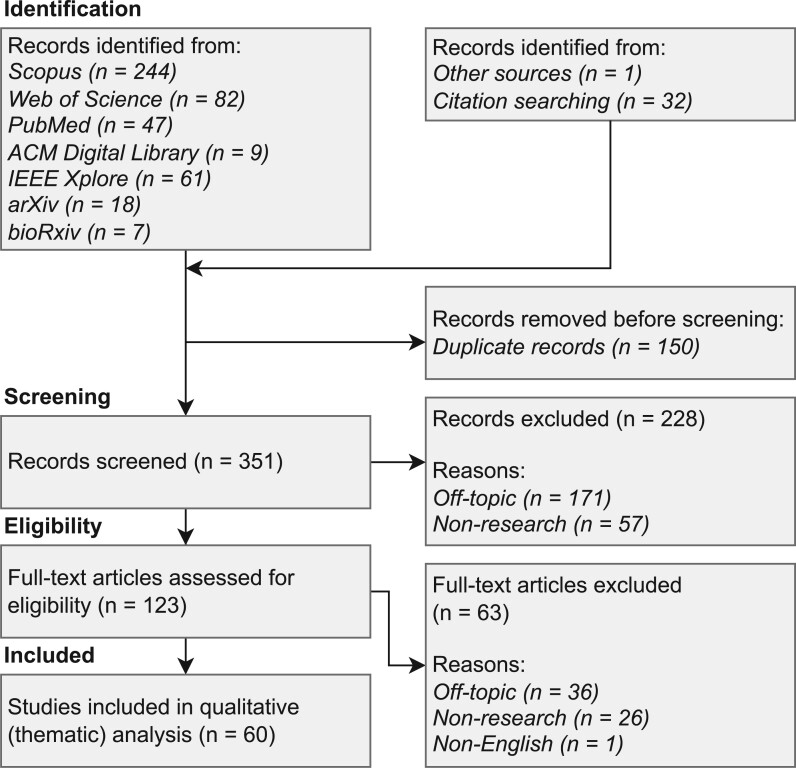
PRISMA flow diagram of the study selection process.

To be eligible for further analysis, publications either describe concepts or solutions based on DLT, tightly integrated with genomic data or with general health data that were applied to genomic data, or discuss ethical, legal, or social implications of DLT in the context of genomics. After removing 150 duplicates, we first screened the titles and abstracts of the remaining 351 articles for eligibility. Screening was performed in parallel by 2 authors. Agreement between the 2 authors was generally very high during the screening process (Cronbach’s Alpha of 0.9). Differences in estimated relevance were discussed with a third author to break ties. Overall, the initial screening resulted in the exclusion of 228 articles. We excluded 171 articles that we deemed off-topic (ie, they had no or only a marginal fit to our topic of interest), and 57 articles that we classified as non-research (eg, white paper, non-peer-reviewed article, news article, conference review, etc.). Next, 2 authors conducted a full-text assessment of the remaining 123 articles. Full-text assessments were again performed in parallel (Cronbach’s Alpha of 0.83) and differences discussed with a third researcher. After excluding another 63 articles (36 were off-topic, 26 were non-research, and one whose full text was not in English), the final sample of relevant publications contained 60 studies. The study selection process is depicted in Figure 2. A full list of relevant studies is included in Supplementary Table S4.

### Data analysis

Following Arksey and O’Malley,^18^ we first collected and coded descriptive data about each included publication. A summary of the collected and coded data is included in Supplementary Table S5. Next, we conducted a thematic analysis following Braun and Clarke,^19^ who propose 6 phases corresponding to following goals: familiarization with data (which we did during the study selection process), initial code generation, theme search, theme review, theme refinement, and reporting. Two authors conducted the initial code generation in parallel and reviewed the results of each other’s work. Afterward, both authors individually collated codes to potential themes and used a mind map in the finalization process (see Supplementary Figure S6). Together, both authors reviewed the potential themes and merged them, resulting in a single hierarchical mind map. With the help of a third author, the themes and the mind map were refined.

## RESULTS

### Descriptive results

**Figure 3.**
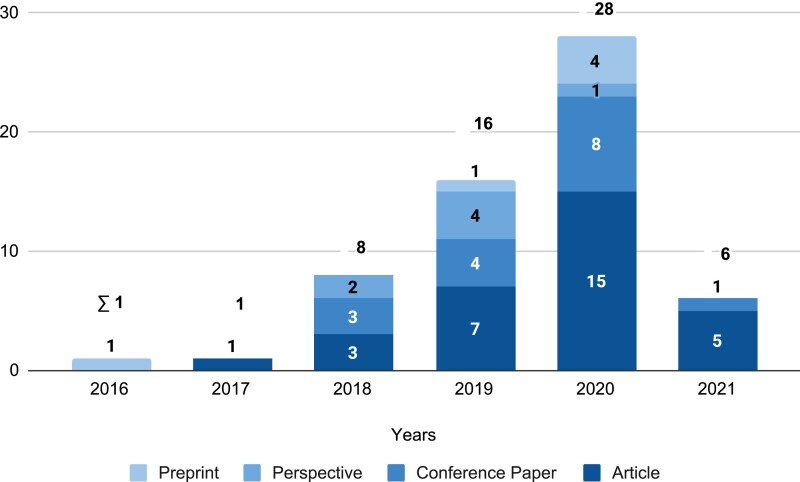
Number of included publications by year and document type.

**Figure 4.**
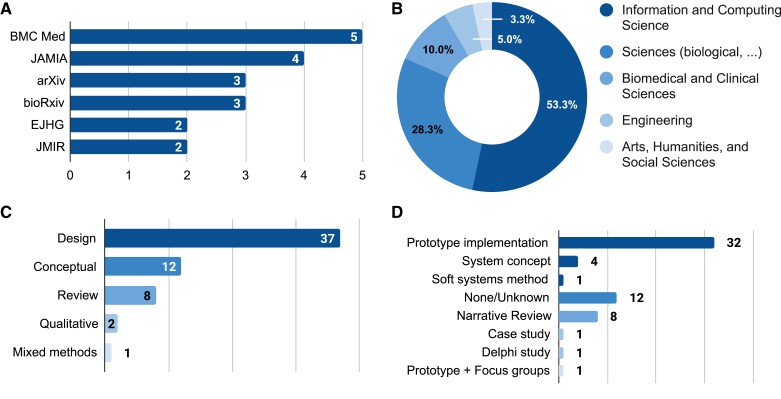
Top-5 outlets (A), overview of scientific disciplines (B), used approaches (C), and employed methods (D) of included publications.

Our search and selection strategy yielded 60 publications published between 2016 and 2021. Overall, we see a steady increase in publications per year apart from 2021 (Figure 3), which is likely due to the search having been conducted midyear. The 60 publications were published in 46 outlets, with the top-6 outlets accounting for over 30% of the total identified publications (Figure 4A). The remaining 40 outlets each only published 1 relevant publication and 1 preprint could not be associated with any outlet. Regarding scientific disciplines, we see that most studies (32) emerged from the information and computing sciences, including medical informatics (Figure 4B); 17 emerged in the sciences (ie, mostly biology-related outlets). Also, 6 studies emerged in the biomedical and clinical sciences, 3 in engineering, and 2 studies stemed from the arts, humanities, and social sciences.

**Figure 5.**
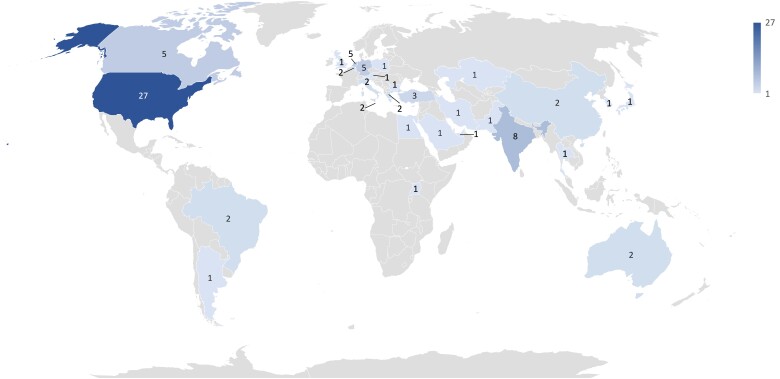
Heat map of publications by countries with at least 1 author of a publication being based in that country. Light blue indicates fewer publications, dark blue more.

Regarding the employed methods, most of the identified studies followed a design approach (*n* = 37), conducting a prototype implementation, creating a system concept, or using the soft systems method (ie, an approach for organizational process modeling, which incorporates the involved stakeholders to solve a problem or improve a process) to develop design principles (Figure 4C and D). Twelve studies were conceptual in nature and provided perspectives, commentary, or a call for research. We also identified 2 qualitative studies (ie, a case study and Delphi study), and 1 mixed methods article that implemented a prototype and subsequently conducted focus groups (Figure 4D). Further, the sample comprised 8 reviews, all of which were narrative reviews (ie, with a select literature and no details on the selection and analysis process). Lastly, as can be seen in Figure 5, most publications were written by at least 1 author based in North America, in particular the United States (27) and Canada (5). Other popular countries lay in Europe such as Germany (5) or the Netherlands (5). Within Asia, India provided the most publications with at least 1 author based in the country (8).

As to the DLT designs scholars based their research on, the 3 most prevalent ones were Multichain (*n* = 8), Hyperledger Fabric (*n* = 7), and Ethereum (*n* = 6), which together make up 40% (Table 2). Overall, the DLT concept of Blockchain shaped most articles’ perspectives (*n* = 58), only 2 papers had a focus on DLT in general. Also noteworthy is the fact that 5 articles focused on custom Blockchain designs, of which 2 only partially resembled Blockchains.

**Table 2.** Overview of DLT concepts and DLT designs researched in literature

DLT concept	DLT design	Count	Share (%)
*DLT in general, no specific focus*		2	3.33
Blockchain	*Not specified*	26	43.33
	BigchainDB	1	1.67
	Consortium Blockchain	2	3.33
	Corda	1	1.67
	Custom Blockchain	3	5.00
	Custom partial Blockchain	2	3.33
	Ethereum	6	10.00
	Exonum	1	1.67
	Hyperledger Fabric	7	11.67
	Hyperledger Indy	1	1.67
	MultiChain	8	13.33
Total		60	100

### Focal research themes

The thematic analysis yielded 7 themes related to DLT in genomics, which we summarize in Table 3. In the following, we describe each theme. A mapping of themes to articles is presented in Supplementary Table S7.

**Table 3.** Overview of identified themes, main findings, and exemplary sources

Theme	Main findings	Exemplary references
Data economy and sharing	DLT can serve as a secure and reliable platform for sharing genetic data DLT enables research data democratization (eg, by breaking up data silos) *Data markets* based on DLT allow for commercialization of personal genetic data DLT creates sharing incentivization through security, privacy, and *monetization* of genetic data (eg, token-based data trading)	^25^ ^,^ ^29^ ^,^ ^33^ ^,^ ^36^ ^,^ ^38^ ^,^ ^39^ ^,^ ^43^ ^,^ ^45^
Data management	DLT can support *data management* by providing governance and access regulation for sharing genetic data *Data access and access control* can be documented on the ledger and automated In *data governance*, DLT ensures data properties such as data quality, data security, data availability, and data ownership	^11^ ^,^ ^22^ ^,^ ^24^ ^,^ ^28^ ^,^ ^29^ ^,^ ^34^ ^,^ ^46–49^
Data protection	DLT’s technical nature allows for protection of sensitive personal and genetic data DLT offers data security features such as data integrity, data immutability, and version history, preventing single points of failure Data transparency creates higher accountability and traceability but may also create data visibility issues (eg, third party access)	^9^ ^,^ ^11^ ^,^ ^15^ ^,^ ^24^ ^,^ ^30^ ^,^ ^31^ ^,^ ^50^
Data storage	Many applications and concepts use DLT as *data storage* for application data and metadata Especially useful when handling big or sensitive data, *off-ledger data storage* refers to storing data externally while keeping only references on the ledger. *On-ledger data storage* describes storing data on the ledger. This concept is feasible for metadata and small or insensitive data The replicated nature of current DLT designs leads to high *storage cost for genetic data*	^11^ ^,^ ^34^ ^,^ ^49^ ^,^ ^51^ ^,^ ^52^
Decentralized data analysis	DLT provides transparency and security in the context of *decentralized data analysis* In decentralized *machine learning*, DLT disintermediates processes of coordination (eg, node enrollment, model synchronization, and leader election) A platform that combines storage and analysis of genetic data is enabled by DLT	^27^ ^,^ ^32^ ^,^ ^40^ ^,^ ^42^ ^,^ ^53–55^
Proof of Useful Work	Proof of Useful Work aims to use DLT nodes’ computational power for computation-intensive tasks in genomics	^12^ ^,^ ^32^
Ethical, legal, and social implications	The use of DLT in genomics bares old and novel ethical, legal, and social implications DLT can foster user privacy but does not solve the risk of reidentification or kinship privacy for genomic data DLT provides user empowerment through self-governance of users’ genetic data DLT-enabled dynamic consent increases users’ control of their data but may be too burdensome Due to its novelty the use of DLT in genomics still lacks regulation Currently, DLT does not solve problems with digital rights and fair use of data DLT cannot solve the lack of diversity in biobanks but may create incentives for minority populations to share their data	^21^ ^,^ ^23^ ^,^ ^24^ ^,^ ^28^ ^,^ ^29^ ^,^ ^32^ ^,^ ^35^ ^,^ ^39^ ^,^ ^46^ ^,^ ^50^ ^,^ ^52^ ^,^ ^55–57^

#### Data economy and sharing

To find efficient ways of sharing genetic data between data providers and data consumers, DLT is often proposed as a platform solution in what some call *data economy*.^22–24^ DLT-based data economies can enable secure^25^^,^^26^ and privacy-aware^27^ data sharing and provide incentives to share one’s data.^22^^,^^28^ While almost every use case of DLT in genomics that was mentioned in the literature facilitates *data sharing*,^29–32^ we further identified 3 specific subthemes in literature: *research data democratization*, *data markets*, and *data sharing incentivization*.

*Research data democratization* refers to enabling the availability of genetic data to anyone interested.^24^^,^^33–35^ Due to its decentralized nature, DLT can encourage research collaboration by breaking up data silos and enable cost-effective real-time workflows between multiple entities (eg, clinical-trial data sharing).^22^^,^^26^^,^^36–38^ Moreover, DLT-based data economies allow individuals to engage with genomics research by contributing their genetic data. Researchers may target individuals with genetic traits of interest directly,^34^^,^^39^ eliminating central custodians.^40^

To commodify such data sharing platforms, several researchers propose DLT-based genetic *data markets*.^13^^,^^41^^,^^42^ Extant literature discusses data platforms such as Encrypgen,^43^ Zenome,^34^ LunaDNA,^43^ or Nebula Genomics,^29^ which utilize DLT to connect data donors and researchers.

DLT-powered data markets also enable various ways of providing *data sharing incentivization* for genetic data.^24^^,^^29^ These data markets allow controlled donation of personal genetic data. Thus, individuals might monetize their data.^39^ Some platforms, such as LifeCode.ai, utilize DLT-enabled token-based data trading (ie, sharing of data is rewarded with crypto currency tokens) to incentivize and compensate for the sharing of genetic data. These tokens can then either be used to purchase further services, such as DNA analysis, or traded for other currencies.^11^^,^^13^^,^^44^ Literature especially mentions the monetization of data as an incentive to participate in research and share personal data.^11^^,^^24^^,^^29^ Further, the benefits of secure^25^^,^^26^ and privacy-aware^28^^,^^45^ data sharing through DLT may also provide additional incentives for sharing one’s personal genetic data.

#### Data management

In our literature review, we identified multiple studies dealing with storing, organizing, and maintaining data. We summarized those findings under the theme *data management*. There, DLT is applied to enhance transparency and security in underlying technical processes. Since genetic data is very sensitive, the thorough management of these data is of paramount importance. Generally, this theme describes access to resources, either by limiting, enabling, enhancing, or documenting it. We identified 2 subthemes, *data access* and *data governance*.

*Data access* largely covers access control and access management utilizing DLT. They relate to authorization and documentation of genetic data access, respectively. Access control also encompasses concepts like participatory access control (ie, data access can be managed by multiple involved entities such as the data donor, their relatives, their healthcare professionals, or researchers), or emergency access (ie, data can be accessed if the data donor loses access or is unable to consent to the data access).^24^^,^^28^ A novel approach to managing shared ownership of genomic data is mentioned by Uribe and Waters.^57^ There, non-fungible tokens (NFTs) are tied to the ownership of genetic information. Since NFTs are noninterchangeable units of data stored on a ledger,^58^ the ownership of genetic data can be managed and even shared depending on the NFT type (eg, ERC-1155). A less strict approach to handling data access to genomic data in a DLT context is access management. Instead of using a ledger or smart contracts to restrict data access, the ledger is used to document it securely. This approach provides less security, but greater transparency. An instrument often used is data access logs, which are managed on a ledger.^46–48^

*Data governance* refers to the use of DLT to ensure data properties such as data quality, data security, or data availability. Through extensive data-management measures, data platforms like Zenome or LifeCODE.ai enforce desired levels of data quality.^34^ This introduces the benefits of data governance into genomic data sharing while relieving data donors of that duty. As an integral aspect of data governance, data ownership describes means by which DLT ensures data control and data sovereignty by the owner (often the donor) themselves.^11^^,^^22^^,^^29^^,^^32^ Due to the decentralized nature of DLT, the technology allows for novel ways of data control (eg, through data markets).^11^^,^^27^^,^^29^ This creates a new model of ownership for genetic data where users hold data sovereignty and consequently are able to govern their data independently.^56^^,^^57^

#### Data protection

Another theme found in literature, namely *data protection*, discusses several technical means DLT can implement to protect sensitive genetic data. In particular, 3 subthemes describing different aspects of protecting genetic data through DLT emerged during our review, *single points of failure*, *data security*, and *data transparency*.

Due to its decentralized nature, DLT can inherently prevent *single points of failure* by distributing data across multiple nodes.^15^^,^^27^ For example, if a centralized biobank loses power it’s data cannot be accessed, so the biobank has a single point of failure. DLT enabled platforms guarantee a more stable information system, with more reliable data availability and sharing, provided the data is stored on-ledger.

Additionally, most papers discuss *data security* features enabled through DLT.^23^^,^^30^^,^^49^ Aspects include data integrity,^31^^,^^59^ data immutability,^9^^,^^60^ and version history.^15^ These aspects allow DLT to ensure data security for genetic data by storing the information on the ledger, which cannot be altered post hoc. Additionally, Dwarna, a DLT system for biobanking introduced by Mamo et al, utilizes pseudonymization and separation of information on-ledger and off-ledger to ensure data security (on- and off-ledger designate storage models used within DLT applications; cf. data storage theme).^50^

Lastly, the *data transparency* subtheme covers 2 aspects of DLT enabled transparency. For one, DLT increases transparency through logging all transactions on the ledger.^9^^,^^56^ This allows for higher accountability and traceability—also for data queries.^11^^,^^45^ At the same time, transparency may also create data visibility issues for sensitive data (or metadata), as third parties may view data stored on the ledger.^24^^,^^49^

#### Data storage

Literature shows that DLT is also being used for storage in genomics. Correspondingly, we identified the theme *data storage* with 3 subthemes: *off-ledger data storage*, *on-ledger data storage*, and *storage cost*.

*Off-ledger data storage* (or off-chain data storage) describes scenarios where only metadata like references, data hashes, or access rights are stored on the ledger while application data is being stored externally. This approach is useful when dealing with big or sensitive data that should not be stored on the ledger due to economical or privacy-related concerns (eg, genetic data). Examples are Zhang et al^49^ who used an off-ledger storage model for large and private data, or Shuaib et al^34^ and Glicksberg et al^51^ who use off-ledger storage for patient and genomic data within cancer treatment. By contrast, *on-ledger data storage* (or on-chain storage) characterizes use cases that store application data within the ledger, improving durability and the possibility of having secure data storage.^15^^,^^52^ However, a negative factor mentioned is the missing ability to correct errors caused by the data-integrity property (ie, because no transaction can be deleted from the ledger, erroneous entries must also stay included).^11^

Due to the highly replicated nature of current DLT designs, resources like computation time and storage are scarce, leading to comparatively high *storage costs*. This is an important factor in designing DLT-based systems that deal with genomic data (or data in general), which tend to be large in size.^34^

On the upside, replication results in high data availability. Since life science data, or genomics data specifically, tend to have big data sizes, the use of on-ledger data storage needs to be balanced carefully (eg, Gürsoy et al^42^).

#### Decentralized data analysis

We further identified a theme concerned with *decentralized data analysis*. It consists of the subthemes *machine learning* and *data analysis*.

In the context of decentralized machine learning approaches (like federated learning) that are being applied to genetic data, the *machine learning* subtheme focuses on how DLT is used to allow decentralized, transparent, and secure process organization and coordination. For example, node enrollment (ie, the process by which a node joins a federated-learning network) allows new nodes to retrieve the shared model (eg, in federated-learning environments, where a single, global machine-learning model is maintained collaboratively) and train it locally with the genetic data in their possession.^53^ After training locally, the model synchronization process, subsequently, allows nodes to contribute their model updates to the shared machine-learning model.^53^^,^^54^ Based on DLT, these processes are designed in a decentralized and secure manner.

More general approaches are described in the theme *data analysis*. An example of which is provided by Zhang et al.^32^ They present a decentralized platform for storage and analysis of genome data for use in genome-wide association studies. In their work, DLT is used as a transparent and integrity-providing log for interactions between data owners and data analysts. Furthermore, genomic data is stored fragmented to minimize the risk of reidentification. There are several other examples of data analysis supported by DLT in literature.^26^^,^^27^^,^^42^^,^^55^^,^^61^

#### Proof of useful work

Whereas newer consensus mechanisms (eg, Proof of Stake) used in DLT designs no longer rely on Proof of Work (PoW) and its resource-demanding hashing,^62^ it is still used for many designs’ consensus (eg, Bitcoin, Ethereum). A modification of PoW that is discussed in some research communities, is *Proof of Useful Work* (PoUW),^12^^,^^32^ which repurposes the computational power used for consensus finding and securing of DLT networks to conduct scientific analyses. Thus, PoUW exhibits similarity to collaborative analyses conducted in distributed-computing projects like SETI@home or Folding@home.^63^ A known DLT design that solves DNA alignment problems as PoUW is Coinami.^12^ Here, high-throughput sequencing reads are mapped to reference genomes. The reads are provided by an authority that also checks the validity of the read mapping results or adjusts the difficulty of the read assignments. Zhang et al^32^ show a similar concept for analysis of gene sequence fragments.

#### Ethical, legal, and social implications

The last theme discusses a variety of ethical, legal, and social implications regarding the use of DLT in genomics. It comprises the 4 subthemes *user privacy*, *user empowerment*, *regulations*, and *social aspects*.

The *user privacy* subtheme largely revolves around the confidentiality of the users’ data, including genomic data stored within the DLT system.^11^^,^^27^^,^^28^^,^^32^^,^^34^^,^^64^ One problem of genomic data mentioned by Zhang et al^32^ is the risk of reidentification, that is, individuals may be identifiable through their unique DNA even when anonymized.^27^^,^^32^ Gene sequence fragmentation, meaning only sharing smaller parts of the DNA, could reduce the risk of reidentification and data privacy in general. Moreover, DLT may pose problems regarding kinship privacy (ie, the privacy of relatives), as genetic information may also identify relatives without their consent.^24^

A potential benefit of DLT in genomics discussed in the literature is *user empowerment*.^15^^,^^21^^,^^34^ DLT allows self-governance of users’ genetic data.^11^^,^^39^ Further, DLT gives users options for consent management. Users know exactly which data sharing they consented to and can withdraw their consent at any time.^22^^,^^30^^,^^39^ This so called dynamic consent is enabled through tracking consent changes on the ledger,^50^ empowering users to share their data while ensuring privacy.^35^ However, some researchers argue that dynamic consent management for genetic data might overwhelm users.^35^

We also identified papers discussing current and future *regulations of DLT in genomics*.^27–29^ Most of these point out that there is still a regulation uncertainty regarding the use of DLT in genomics^24^^,^^36^ and call for more legal guidelines.^28^^,^^57^ One problem brought up by Gürsoy et al^46^ is the right to be forgotten problem, which is especially serious for genetic data, as it can uniquely identify a human. Data cannot be deleted and is stored on the ledger forever, potentially contradicting the right to be forgotten.^36^^,^^46^

Lastly, several *social aspects* for DLT in genomics were also brought up in literature. Emphasizing the need for digital rights in genomics, Chavali et al^52^ and Uribe and Waters^57^ propose using DLT technologies such as NFTs to enforce personal control over one’s digital genomic data.^52^^,^^57^ Moreover, another social aspect brought up by Racine is the potential lack of diversity in genomic biobanks, which may have negative healthcare consequences for certain groups.^35^ Literature also deals with the so-called free-riding problem (ie, the unfair use of publicly available information such as genetic data in biobanks).^23^

## DISCUSSION

The present literature review yielded 7 themes on DLT in genomics, highlighting manifold applications of DLT in the context of genomics. Since research on DLT in general and research on its applications in genomics in particular is still in its early stages, there are still many open questions and knowledge gaps. To answer our second research question, we thus discuss several avenues for future research on DLT in genomics and propose concrete research directions. We also provide exemplary research questions for each research direction in the Supplementary Material S8.

### Directions for future research

#### Direction 1: Explore opportunities for the application of DLT concepts other than Blockchain

Although there are several DLT concepts (eg, Blockchain, TDAG, and BlockDAG) and DLT designs (eg, Bitcoin, Ethereum, etc.),^1^ our review revealed that extant research only focuses on the Blockchain concept and a few associated DLT designs (see Table 2). This is not surprising, as to date Blockchain is the most popular DLT concept used in practice and investigated in research. Adding to this, the terms Blockchain and DLT are often used synonymously, although Blockchain is a more specific concept than DLT. This may be due to convenience or a lack of understanding of the difference. We believe it is important to investigate other DLT concepts beyond Blockchain and see if they can help address pertinent issues outlined above regarding data management, data protection, or data storage and the use of Blockchain in genomics. Especially, performance is a feature where non-Blockchain DLT concepts have great potential, since they are not limited by a strictly linear ledger structure that needs to be maintained by consensus mechanisms that often incur computation and communication cost for participating nodes. To fill existing knowledge gaps, future research should therefore explore opportunities and risks for other DLT concepts in genomics and help develop a more differentiated view on the matter.

#### Direction 2: Investigate people’s attitudes and behaviors regarding the commodification of genetic data through DLT-based genetic data markets

Genetic data markets based on DLT and monetization of genetic data are hot topics.^13^^,^^15^^,^^24^^,^^26^^,^^34^^,^^38^^,^^41–44^^,^^49^^,^^55^ In our review, we identified mostly technical and conceptual papers with varying degrees of maturity—but generally, a lot of hope is put into this novel technology. Several biotech startups are already building and operating genetic data markets based on DLT. These startups openly use DLT in their marketing strategies since it is widely recognized as a technology associated with trust. However, data markets are a very recent cultural phenomenon; it is not clear how people will react to them and how much of that reaction is shaped by the use of DLT. Past research has shown that people might see the commodification of genetic data as problematic. Thus, we feel there is a need to also explore whether and how DLT-based data markets foster the commodification of genetic data, potential implications of this, and people’s attitudes and actual behaviors regarding such (DLT-based) data markets.

#### Direction 3: Examine opportunities for joint consent management via DLT

Although genetic data inherently raise interdependent privacy issues (ie, issues of privacy affecting more than 1 person), extant research on (DLT-based) consent management focuses on privacy and consent on a per individual basis. Research in other related areas has long discussed whether genetic data should be considered personal or familial data and proposed joint account or consent models.^65–67^ However, for legal and ethical but also practical reasons, such joint account models have thus far not been put into practice. Given DLT’s ability to establish a system of trust in which pseudonymous but authentic actions are possible without the need for intermediaries, as well as its automation capabilities through smart contracts, we believe that DLT could help to tackle some of these issues. Although there is some research in that direction,^68^ we think it is worth to further explore how DLT can enable joint consent management. In particular, research should investigate how joint consent management schemes can be translated into DLT-based systems and how such systems should be designed.

#### Direction 4: Investigate and evaluate data storage models appropriate for DLT

Many concepts and implementations utilize DLT to store genomics data either off-ledger or on-ledger. Most current DLT systems are highly replicated; thus, computations and storage are expensive operations. When storing genomic data on-ledger, the DLT architecture can inherently prevent single points of failure.^15^^,^^27^ While scarce, research supporting on-ledger storage suggests the use of compression algorithms reducing size of genetic data.^61^ Due to the ever-increasing amounts of generated data and increasing size for single DNA sequences, however, this may only be a temporary solution. How to use DLT systems effectively regarding data storage is not broadly understood. Hence, off-ledger and on-ledger storage as well as means of efficiently storing genetic data should be explored and evaluated extensively.

#### Direction 5: Research the regulation-compliant use of DLT in healthcare information systems

Genetic data, and medical data in general, contain personal and very sensitive information. Accordingly, information systems dealing with such data need to comply with privacy regulations (eg, HIPAA, GDPR). Due to their transparent nature, most DLT systems do not lend themselves to storing such sensitive data. For example, genetic data stored on-ledger may contradict a user’s right to be forgotten.^36^^,^^46^ This problem also expands to metadata such as user’s personal information and dynamic consent changes, which need to be deleted upon request to comply with current privacy regulations.^50^ It is unclear whether and how DLT can benefit medical information systems falling under such regulations. With strong assumptions on the purpose of use, there are concepts that aim to circumvent these issues. By relying on federated learning to analyze the genetic data on site, researchers have used DLT to only manage the organization and coordination of processes within decentralized data analysis omitting sharing and external storage of genetic data and most metadata. As our review shows, DLT systems are also being considered for sharing of electronic health records with genetic data being just 1 type of health information stored.^22^^,^^41^^,^^56^^,^^69^ Further research is needed to explore the use of DLT in compliance with different privacy regulations not only in the context of genomics but also for health care and medicine in general.

#### Direction 6: Investigate alternative consensus mechanisms based on proof of useful work

Given the perception of PoW being a waste of a DLT network’s computational power (eg, references^70–72^) alternate consensus mechanisms that build on PoUW seem like a promising approach to redirect and utilize the computational power of a DLT network for other, meaningful purposes. Despite first proposals for the use of PoUW in the genomics context,^12^ there still remain many open questions regarding its specific advantages and disadvantages or suitable tasks for PoUW in this context. For example, to create and audit genomic analysis tasks for use in PoUW-based consensus mechanisms, an authority is needed. Currently, these authorities are central bodies and thus incur centralization issues in the decentralized DLT network. To make sense from a DLT perspective, alternative consensus mechanisms should not increase centralization, all participants should be equal, and interactions within the network should not be based solely on trust (eg, trusting that an authority is performing its job honestly). It is unclear whether PoUW can be introduced without centralization (ie, whether centralization is a systemic issue inherent in PoUW itself and independent of the chosen PoUW approach). Thus, future research should explore methods to provide PoUW-based DLT systems with tasks and assess the results without incurring centralization issues. Research should also assess how to effectively utilize PoUW in genomics in general and attain knowledge on what kind of genetic analysis tasks are suited for use in PoUW-based consensus mechanisms.

#### Direction 7: Explore DLT-enabled approaches for the protection of genetic data ensuring user privacy

As with other novel technologies, DLT promises to offer a wide range of unique data-protection and security features such as data integrity, tamper resistance, and version history.^9^^,^^15^^,^^31^ However, especially data transparency might be a threat to user privacy. Although this DLT inherent feature increases data protection by adding all transactions to the ledger,^56^ associated data-visibility issues may enable third parties to view or even access sensitive data such as genetic data, thus affecting user privacy.^24^ While some DLT systems such as Dwarna try to mitigate this problem through information separation and therefore data pseudonymization,^50^ it is debatable whether this approach is sufficient for application in genomics. As previous research has shown, access to genetic data without any additional metadata may be ample to uniquely identify a person’s identity.^73^ With DLT being a novel technology, many social and ethical implications on user privacy especially in the field of genomics are not yet well understood. Therefore, further research should explore methods for protecting genetic data with DLT while ensuring user privacy. Also, socio-technical aspects (eg, key management) arising when protecting genetic data with DLT should be investigated and understood.

### Limitations

Our research is not without limitations. First, the thematic analysis method is somewhat subjective. We addressed this concern by including multiple researchers in the analyses. Second, the application of DLT in genomics defines a nascent and interdisciplinary research stream with a rapidly growing number of publications from different research areas. Owing to that fact, our reviews recency may decline over time, and it may be necessary to repeat the review after some time. Third, we only considered research articles. This excluded white papers which, for example, cover genomic data markets in more detail. Nevertheless, we are confident to have covered a broad range of aspects related to DLT in genomics, including data markets.

## CONCLUSION

In this study, we investigated extant literature on DLT in genomics by means of a scoping review and thematic analysis. We identified 7 focal research themes namely, *data economy and sharing, data management, data protection, data storage, proof of useful work,* and *ethical, legal, and social implications*. While research on DLT in genomics is currently growing rapidly, many problems remain unanswered. As a contribution to research and practice, we developed several future research directions with the aim of paving the way for the adoption of DLT in genomics.

## FUNDING

The present contribution is supported by the Helmholtz Association under the joint research school “HIDSS4Health—Helmholtz Information and Data Science School for Health.”

## AUTHOR CONTRIBUTIONS

Author contributions according to the CRediT—Contributor Roles Taxonomy are as follows. MB: conceptualization; data curation; formal analysis; investigation; methodology; visualization; writing original draft; writing, review, and editing. PAT: conceptualization; data curation; formal analysis; investigation; methodology; visualization; writing original draft; writing, review, and editing. ST: conceptualization; funding acquisition; investigation; methodology; project administration; supervision; visualization; writing original draft; writing, review, and editing. MS: funding acquisition; supervision; writing, review, and editing. BB: funding acquisition; supervision; writing, review, and editing. AS: funding acquisition; resources; supervision; writing original draft; writing, review, and editing.

## SUPPLEMENTARY MATERIAL

Supplementary material is available at *Journal of the American Medical Informatics Association* online.

## Supplementary Material

ocac077_supplementary_data
