# Supplementary material for: A scoping review of distributed ledger technology in genomics: thematic analysis and directions for future research
Source: J Am Med Inform Assoc. 2022 May 20;29(8):1433–44. doi: 10.1093/jamia/ocac077 (PMC9277639; doi:10.1093/jamia/ocac077)
Supplement: ocac077_supplementary_data [file ocac077_supplementary_data.zip › S1_DLT_Background.pdf]

S1: BACKGROUND ON DISTRIBUTED LEDGER TECHNOLOGY

Distributed Ledger Technology (DLT) is an umbrella term for a nascent class of technologies (including blockchain) that enable the operation of a highly available, append-only distributed database (referred to as a distributed ledger) in untrustworthy environments.[1] In a DLT network, various storage and computing devices, called nodes, maintain local replications of the ledger.[2] To transfer or append data to the ledger, DLT makes use of transactions.[2] These transactions are created by external users of a DLT network or by nodes themselves and contain metadata (e.g., transaction senders and recipients, timestamps) and application data, often being digital representations of certain assets (e.g., coins for a cryptocurrency).[3] Since individual nodes in a DLT network maintain local replications of the distributed ledger, all nodes must be synchronized and agree on a common state of the distributed ledger in order to establish consistency.[2] The negotiation of a common state between nodes (i.e., decide which transactions are accepted and which are rejected by the network) is thereby managed by a set of protocols and algorithms, so-called consensus mechanisms.[2, 4] They build on trust models and are designed to overcome Byzantine failures like unreachable nodes, network delays, or malicious behavior of some nodes, which are characteristic for untrustworthy environments.[5]

DLT comprises various DLT concepts and DLT designs with different characteristics.[6] Thereby, DLT concepts are abstract, high-level descriptions of the basic structure and functioning of DLT designs.[2] The most prominent DLT concept blockchain, for example, describes the organization of transactions in the form of a linked list (i.e., a chain) of blocks, with each block containing multiple transactions. Other DLT concepts use different organizing structures that do not form chains of blocks or do not make use of blocks at all. The DLT concept BlockDAG, for instance, organizes transactions in blocks that form a directed acyclic graph (DAG), whereas the DLT concept TDAG organizes transactions in a DAG without first combining them into blocks. Figure S1.1 shows an overview of DLT concepts and which DLT designs they relate to.

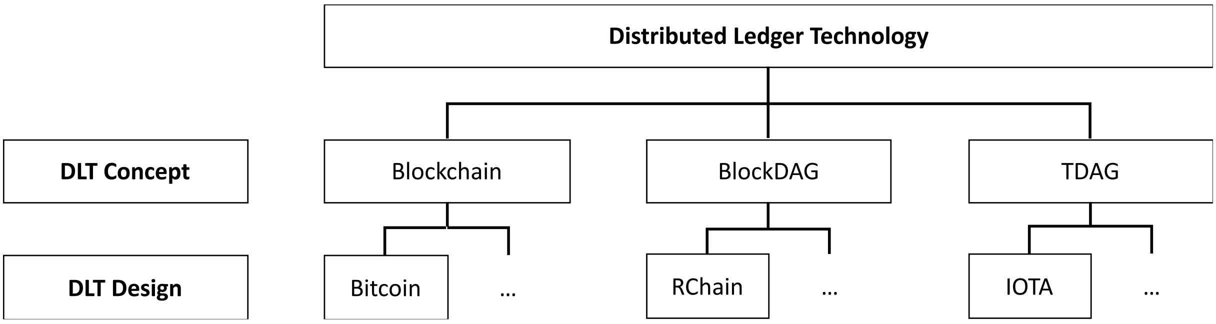

Figure S1.1. Structured presentation of terminology in the context of DLT. Adapted from Kannengießer et al.[2]

By contrast, DLT designs (e.g., Bitcoin) are instances of DLT concepts specifying concrete values and processes for inherent DLT characteristics such as a maximum block size (i.e., number of transactions per block) or consensus mechanisms.[2] As such, there are important differences between DLT designs that make individual DLT designs more suitable for some applications than for others.[2] DLT designs can, for example, differ in the choice of consensus

mechanism. This has far-reaching consequences and influences, among other things, performance, scalability (i.e., the maximum possible number of nodes in the network), security guarantees, degree of decentralization, and also energy consumption.[7] Toward that end, consensus mechanisms based on Proof of Work (PoW) are computationally expensive, making it difficult to exploit by attackers and providing the highest security guarantees among consensus mechanisms, but also to high energy consumption.[7, 8] However, not all applications of DLT require such level of security, which is why other DLT designs with different, less computationally intensive consensus mechanisms may also be suitable for use. Another feature that characterizes many modern DLT designs are so-called smart contracts. These are applications that are executed in a distributed manner on all nodes in the DLT network. This approach offers higher transparency and higher security guarantees compared to programs that are executed on central servers (especially with regard to data integrity and availability). Ethereum was the first DLT design to support smart contracts.[3]

With regard to DLT applications, tokens (in the form of cryptocurrencies) were the first application area. Tokens are transferable units of information. Cryptographic processes ensure that they are not copied but must change hands during transfer.[9] We differentiate between fungible tokens (i.e., tokens that cannot be distinguished from one another) and non-fungible tokens (NFTs). The function of tokens is to represent real-world or digital entities (e.g., currencies, real estate, rights, etc.).[10] But DLT applications are not only found in the initial area of finance but are being used in a wide variety of industries. There are applications in healthcare, manufacturing, the arts, and administration, to name a few domains.[11-14]

REFERENCES

1. Zhang K, Jacobsen H-A. Towards Dependable, Scalable, and Pervasive Distributed Ledgers with Blockchains (Technical Report). 2018.
2. Kannengießer N, Lins S, Dehling T, Sunyaev A. Trade-Offs between Distributed Ledger Technology Characteristics. ACM Computing Surveys 2020;**53**(2):1-37 doi: 10.1145/3379463.
3. Buterin V. A Next-Generation Smart Contract and Decentralized Application Platform. white paper 2014;**3**(37).
4. Lashkari B, Musilek P. A Comprehensive Review of Blockchain Consensus Mechanisms. IEEE Access 2021;**9**:43620-52.
5. Shostak R, Pease M, Lamport L. The Byzantine Generals Problem. ACM Transactions on Programming Languages and Systems 1982;**4**(3):382-401.
6. Kannengießer N, Lins S, Dehling T, Sunyaev A. What Does Not Fit Can Be Made to Fit! Trade-Offs in Distributed Ledger Technology Designs. Proceedings of the Annual Hawaii International Conference on System Sciences, 2019:7069-78.
7. An Overview of Blockchain Technology: Architecture, Consensus, and Future Trends. 2017 IEEE international congress on big data (BigData congress); 2017. IEEE.
8. On the Security and Performance of Proof of Work Blockchains. Proceedings of the 2016 ACM SIGSAC conference on computer and communications security; 2016.
9. Hülsemann P, Tumasjan A. Walk This Way! Incentive Structures of Different Token Designs for Blockchain-Based Applications. 2019.
10. Non-Fungible Tokens (Nft)-Innovation Beyond the Craze. 5th International Conference on Innovation in Business, Economics and Marketing Research; 2021.
11. Blockchain Technology in Healthcare: A Systematic Review. Healthcare; 2019. Multidisciplinary Digital Publishing Institute.
12. Leng J, Ruan G, Jiang P, et al. Blockchain-Empowered Sustainable Manufacturing and Product Lifecycle Management in Industry 4.0: A Survey. Renewable and sustainable energy reviews 2020;**132**:110112.
13. Trautman LJ. Virtual Art and Non-Fungible Tokens. Available at SSRN 3814087 2021.

14. Moura LMFd, Brauner DF, Janissek-Muniz R. Blockchain and a Technological Perspective for Public Administration: A Systematic Review. Revista de Administração Contemporânea 2020;**24**:259-74.
