# Supplementary material for: A scoping review of distributed ledger technology in genomics: thematic analysis and directions for future research
Source: J Am Med Inform Assoc. 2022 May 20;29(8):1433–44. doi: 10.1093/jamia/ocac077 (PMC9277639; doi:10.1093/jamia/ocac077)
Supplement: ocac077_supplementary_data [file ocac077_supplementary_data.zip › S2_Table_of_identified_reviews.pdf]

Table S2. List of ten identified reviews that address the application of distributed ledger technology in genomics

Review	Type	Scientific Field	Summary
Engelhardt et al. [1]	Narrative review	Information and Computing Science and Technology	Overview of applications, opportunities, and challenges of Blockchain technology in healthcare.
Chavali et al. [2]	Narrative review	Sciences (mathematical, physical, biological)	Overview of opportunities and challenges of Blockchain technology in life sciences, pharmacy, and agriculture.
Dimitrov et al. [3]	Narrative review	Information and Computing Science and Technology	Overview of digital platforms based on Blockchain technology that allow for interaction between healthcare data providers.
Justinia et al. [4]	Narrative review	Information and Computing Science and Technology	Description and synthesis of real-world use-case scenarios for Blockchain technology in healthcare and biomedical practice.
Jung et al. [5]	Narrative review	Medical and Health Sciences	Overview of applications, opportunities, and challenges of Blockchain technology in genomics.
Ullah et al. [6]	Narrative review	Information and Computing Science and Technology	Overview of applications and associated organizations, challenges, and future perspectives of Blockchain technology in healthcare and medicine.
Chattu et al. [7]	Narrative review	Medical and Health Sciences	Overview of the use of precision medicine in sleep medicine through the application of AI and Blockchain technology.
Dedeturk et al.[a] [8]	Critical review	Sciences (mathematical, physical, chemical, earth, environmental, biological, agricultural)	Overview of opportunities, challenges, and applications of Blockchain technology in genomics and healthcare in research and practice.
Velmovitsky et al. [9]	Narrative review	Medical and Health Sciences	Overview of challenges in healthcare and associated applications of Blockchain technology in practice.
Alghazwi et al.[b] [10]	Scoping review	Information and Computing Science and Technology	Overview of opportunities, challenges, and applications of Blockchain technology in genomics in research and practice.
Beyene et al. (this study)	Scoping review	Information and Computing Science and Technology	Overview of recurring research themes in the literature on distributed ledger technology in genomics. Discussion of several future research directions.

[a] Research article not included in the literature review and analysis, because published after the search was conducted

[b] Preprint not included in the literature review and analysis, because published after the search was conducted

REFERENCES

1. Engelhardt MA. Hitching Healthcare to the Chain: An Introduction to Blockchain Technology in the Healthcare Sector. Technology Innovation Management Review 2017;**7**(10).

2. Chavali LN, Prashanti NL, Sujatha K, Rajasheker G, Kavi Kishor PB. The Emergence of Blockchain Technology and Its Impact in Biotechnology, Pharmacy and Life Sciences. Current Trends in Biotechnology and Pharmacy 2018;**12**(3):304-10.
3. Dimitrov DV. Blockchain Applications for Healthcare Data Management. Healthc Inform Res 2019;**25**(1):51-56 doi: 10.4258/hir.2019.25.1.51.
4. Justinia T. Blockchain Technologies: Opportunities for Solving Real-World Problems in Healthcare and Biomedical Sciences. Acta Inform Med 2019;**27**(4):284-91 doi: 10.5455/aim.2019.27.284-291.
5. Jung T, Leu R. Blockchain’s Potential to Address Issues in Genomics Research and How It Is Being Used Today. University of Western Ontario Medical Journal 2020;**88**(S):13-15 doi: 10.5206/uwomj.v88iS.8233.
6. Ullah HS, Alsam S, Arjomand N. Blockchain in Healthcare and Medicine: A Contemporary Research of Applications, Challenges, and Future Perspectives. https://arxiv.org/abs/2004.06795, 2020:16.
7. Chattu VK, Sunil TS, Santaji S, et al. Precision Medicine Meets Sleep Medicine: How Can Sleep Health Aid to Reduce the Preventable Burden of Non-Communicable Diseases? Sleep and Vigilance 2021;**5**(2):179-88 doi: 10.1007/s41782-021-00149-1.
8. Dedeturk BA, Soran A, Bakir-Gungor B. Blockchain for Genomics and Healthcare: A Literature Review, Current Status, Classification and Open Issues. PeerJ 2021;**9**:e12130.
9. Velmovitsky PE, Bublitz FM, Fadrique LX, Morita PP. Blockchain Applications in Health Care and Public Health: Increased Transparency. JMIR Med Inform 2021;**9**(6):e20713 doi: 10.2196/20713.
10. Alghazwi M, Turkmen F, van der Velde J, Karastoyanova D. Blockchain for Genomics: A Systematic Literature Review. arXiv preprint arXiv:2111.10153 2021.
