# Supplementary material for: A scoping review of distributed ledger technology in genomics: thematic analysis and directions for future research
Source: J Am Med Inform Assoc. 2022 May 20;29(8):1433–44. doi: 10.1093/jamia/ocac077 (PMC9277639; doi:10.1093/jamia/ocac077)
Supplement: ocac077_supplementary_data [file ocac077_supplementary_data.zip › S4_Table_of_relevant_articles.pdf]

Table S4. List of the 60 relevant articles identified per our literature review

Authors	Year	Outlet	Document Type	Title	Scientific Field	Approach	Method	DLT Concept / DLT Design	Authors' Countries
Ileri AM, Ozercan HI, Gundogdu A, et al.	2016	arXiv	Preprint	Coinami: a cryptocurrency with DNA sequence alignment as proof-of-work	Information and Computing Science	Design	Prototype implementation	Blockchain / Custom Blockchain	Turkey
Engelhardt, M	2017	Technology Innovation Management Review	Article	Hitching Healthcare to the Chain: An Introduction to Blockchain Technology in the Healthcare Sector	Arts, Humanities, and Social Sciences	Review	Narrative review	Blockchain / -	Canada
Chavali LN, Prashanti NL, Sujatha K, et al.	2018	Current Trends in Biotechnology and Pharmacy	Article	The emergence of blockchain technology and its impact in biotechnology, pharmacy and life sciences	Sciences (agricultural, biological, chemical, …)	Review	Narrative review	Blockchain / -	India
Dambrot SM	2018	2018 9th IEEE Annual Ubiquitous Computing, Electronics and Mobile Communication Conference, UEMCON 2018	Conference Paper	ReGene: Blockchain backup of genome data and restoration of pre-engineered expressed phenotype	Information and Computing Science	Design	System concept	Blockchain / -	USA
Gökalp E, Gökalp MO, Çoban S, et al.	2018	Lecture Notes in Business Information Processing	Conference Paper	Analysing opportunities and challenges of integrated blockchain technologies in healthcare	Information and Computing Science	Design	System concept	Blockchain / -	Turkey
Grishin D, Obbad K, Estep P, et al.	2018	Blockchain in Healthcare Today	Article	Accelerating genomic data generation and facilitating genomic data access using decentralization, privacy-preserving technologies and equitable compensation	Information and Computing Science	Conceptual	None/unknown	Blockchain / Exonum	USA

Authors	Year	Outlet	Document Type	Title	Scientific Field	Approach	Method	DLT Concept / DLT Design	Authors' Countries
Langley PC, Martin RE	2018	INNOVATIONS in pharmacy	Perspective	If You Build it Will They Come? Patients, Providers and Blockchains in Health Technology Assessment	Biomedical and Clinical Sciences	Conceptual	None/unknown	Blockchain / -	USA
Lee SJ, Cho GY, Ikeno F, et al.	2018	Applied Sciences (Switzerland)	Article	BAQALC: Blockchain Applied Lossless EfficientTransmission of DNA Sequencing Data for NextGeneration Medical Informatics	Sciences (agricultural, biological, chemical, …)	Design	Prototype implementation	Blockchain / -	Republic of Korea, USA
Ozercan HI, Ileri AM, Ayday E, et al.	2018	Genome Research	Perspective	Realizing the potential of blockchain technologies in genomics	Sciences (agricultural, biological, chemical, …)	Conceptual	None/unknown	Blockchain / -	Turkey USA
Zhang X, Sharma R, Wingreen S	2018	Proceedings of the 22nd Pacific Asia Conference on Information Systems - Opportunities and Challenges for the Digitized Society: Are We Ready?, PACIS 2018	Conference Paper	Block-chaining in Precision HealthCare: A design research approach	Information and Computing Science	Design	Prototype implementation	Blockchain / -	New Zealand
Ahmed E, Shabani M	2019	frontiers in Genetics	Perspective	DNA Data Marketplace: An Analysis of the Ethical Concerns Regarding the Participation of the Individuals	Sciences (agricultural, biological, chemical, …)	Conceptual	None/unknown	Blockchain / -	Belgium, Egypt
Akshayaa S, Vidhya R, Krishnan Namboori PK, et al.	2019	Proceedings of the 3rd International Conference on Computing Methodologies and Communication, ICCMC 2019	Conference Paper	Exploring pain insensitivity inducing gene ZFHX2 by using deep convolutional neural network	Information and Computing Science	Design	Prototype implementation	Blockchain / -	India

Authors	Year	Outlet	Document Type	Title	Scientific Field	Approach	Method	DLT Concept / DLT Design	Authors' Countries
Carlini R, Carlini F, Dalla Palma S, et al.	2019	CEUR Workshop Proceedings	Conference Paper	Genesy: A blockchain-based platform for DNA sequencing	Information and Computing Science	Design	Prototype implementation	Blockchain / Hyperledger Fabric	Italy, Netherlands
Dimitrov DV	2019	Healthcare Informatics Research	Article	Blockchain applications for healthcare data management	Information and Computing Science	Review	Narrative review	Blockchain / -	Bulgaria
Iyer V, Hima Vyshnavi AM, Iyer S, et al.	2019	2019 IEEE Bombay Section Signature Conference, IBSSC 2019	Conference Paper	An AI driven Genomic Profiling System and Secure Data Sharing using DLT for cancer patients	Information and Computing Science	Design	Prototype implementation	Blockchain / Corda	India
Jin XL, Zhang M, Zhou Z, et al.	2019	Journal of Medical Internet Research	Article	Application of blockchain platform to manage and secure personal genomic data: A case study of lifecode.AI in China	Information and Computing Science	Qualitative	Case Study	Blockchain / Ethereum	China
Justinia T	2019	Acta Informatica Medica	Article	Blockchain technologies: Opportunities for solving real-world problems in healthcare and biomedical sciences	Information and Computing Science	Review	Narrative review	Blockchain / -	Saudi Arabia
Kuo TT, Gabriel RA, Ohno-Machado L	2019	Journal of the American Medical Informatics Association	Article	Fair compute loads enabled by blockchain: Sharing models by alternating client and server roles	Information and Computing Science	Design	Prototype implementation	Blockchain / MultiChain	USA
Mackey TK, Kuo TT, Gummadi B, et al.	2019	BMC Medicine	Perspective/Opinions/Views	Fit-for-purpose?' - Challenges and opportunities for applications of blockchain technology in the future of healthcare	Biomedical and Clinical Sciences	Conceptual	None/unknown	Blockchain / -	USA
Preethi V, Surve S	2019	International Journal of Engineering and Advanced Technology	Article	Blockchain enabled DNA banking and comparative analysis using hyperledger fabric	Engineering	Design	Prototype implementation	Blockchain / Hyperledger Fabric	India

Authors	Year	Outlet	Document Type	Title	Scientific Field	Approach	Method	DLT Concept / DLT Design	Authors' Countries
Shabani M	2019	Journal of the American Medical Informatics Association	Perspective	Blockchain-based platforms for genomic data sharing: a de-centralized approach in response to the governance problems?	Information and Computing Science	Conceptual	None/unknown	Blockchain / -	Belgium
Sharma R, Zhang C, Wingreen SC, et al.	2019	Industrial Management and Data Systems	Article	Design of Blockchain-based Precision Health-Care Using Soft Systems Methodology	Information and Computing Science	Design	Prototype implementation	Blockchain / Hyperledger Fabric	New Zealand, USA
Venner E, Murugan M, Hale W, et al.	2019	Journal of the American Medical Informatics Association	Article	ARBoR: an identity and security solution for clinical reporting	Information and Computing Science	Design	Prototype implementation	Blockchain / Custom (Partial) Blockchain	USA
Zhang Y, Zhao X, Li X, et al.	2019	WSDM '19: Proceedings of the Twelfth ACM International Conference on Web Search and Data Mining	Conference Paper	Enabling Privacy-preserving Sharing of Genomic Data for GWASs in Decentralized Networks	Information and Computing Science	Design	Prototype implementation	Blockchain / Custom Blockchain	Australia
Zhavoronkov A, Church G	2019	Trends in Molecular Medicine	Perspective/Opinions/Views	The Advent of Human Life Data Economics	Biomedical and Clinical Sciences	Conceptual	None/unknown	Blockchain / -	China, USA
Zimmerman N, Tatonetti NP, Dudley JT	2019	N/A	Preprint	A MARKETPLACE FORHEALTH: OPPORTUNITIES ANDCHALLENGES FORBIOMEDICALBLOCKCHAINS	Sciences (agricultural, biological, chemical, …)	Conceptual	None/unknown	Blockchain / -	USA
Carlini F, Carlini R, Palma SD, et al.	2020	2020 Seventh International Conference on Software Defined Systems (SDS)	Conference Paper	The Genesy Model for a Blockchain-based Fair Ecosystem of Genomic Data	Information and Computing Science	Design	Prototype implementation	Blockchain / Hyperledger Fabric	Italy, Netherlands

Authors	Year	Outlet	Document Type	Title	Scientific Field	Approach	Method	DLT Concept / DLT Design	Authors' Countries
Chernomoretz A, Balparda M, Grutta LL, et al.	2020	bioRxiv	Article	GENis, an open-source multi-tier forensic DNA information system	Sciences (agricultural, biological, chemical, …)	Design	Prototype implementation	Blockchain / -	Argentina
Evangelatos N, Upadya SP, Venne J, et al.	2020	OMICS	Article	Digital Transformation and Governance Innovation for Public Biobanks and Free/Libre Open Source Software Using a Blockchain Technology	Sciences (agricultural, biological, chemical, …)	Design	Prototype implementation	Blockchain / Custom (Partial) Blockchain	Germany, India, Netherlands
Glicksberg BS, Burns S, Currie R, et al.	2020	Journal of Medical Internet Research	Article	Blockchain-authenticated sharing of genomic and clinical outcomes data of patients with cancer: A prospective cohort study	Information and Computing Science	Design	Prototype implementation	Blockchain / Ethereum	USA
Gürsoy G, Bjornson R, Green ME, et al.	2020	BMC Medical Genomics	Article	Using blockchain to log genome dataset access: efficient storage and query	Sciences (agricultural, biological, chemical, …)	Design	Prototype implementation	Blockchain / MultiChain	USA
Gürsoy G, Brannon CM, Gerstein M	2020	BMC Medical Genomics	Article	Using Ethereum blockchain to store and query pharmacogenomics data via smart contracts	Sciences (agricultural, biological, chemical, …)	Design	Prototype implementation	Blockchain / Ethereum	USA
Gürsoy G, Brannon CM, Wagner S, et al.	2020	bioRxiv	Preprint	Storing and analyzing a genome on a blockchain	Sciences (agricultural, biological, chemical, …)	Design	Prototype implementation	Blockchain / MultiChain	USA
Hendricks-Sturrup RM, Lu CY	2020	SAGE Open Medicine	Article	What motivates the sharing of consumer-generated genomic information?	Biomedical and Clinical Sciences	Conceptual	None/unknown	Blockchain / -	USA
Jung T, Leu R	2020	University of Western Ontario Medical Journal	Article	Blockchain's potential to address issues in genomics research and how it is being used today	Biomedical and Clinical Sciences	Review	Narrative review	Blockchain / -	Canada

Authors	Year	Outlet	Document Type	Title	Scientific Field	Approach	Method	DLT Concept / DLT Design	Authors' Countries
Kuo TT, Gabriel RA, Cidambi KR, et al.	2020	Journal of the American Medical Informatics Association	Article	EXpectation Propagation LOgistic REgRession on permissioned blockCHAIN (ExplorerChain): Decentralized online healthcare/genomics predictive model learning	Information and Computing Science	Design	Prototype implementation	Blockchain / MultiChain	USA
Kuo TT	2020	JAMIA Open	Article	The anatomy of a distributed predictive modeling framework: online learning, blockchain network, and consensus algorithm	Information and Computing Science	Design	Prototype implementation	Blockchain / MultiChain	USA
Lemieux VL	2020	frontiers in Blockchain	Article	Having our 'omic' cake and eating it too: Evaluating UserResponse to using Blockchain Technologyfor Private & Secure Health Data Management and Sharing	Information and Computing Science	Mixed methods	Prototype implementation + Focus groups	Blockchain / Hyperledger Indy	Canada
Ma S, Cao Y, Xiong L	2020	BMC Medical Genomics	Article	Efficient logging and querying for blockchain-based cross-site genomic dataset access audit	Sciences (agricultural, biological, chemical, …)	Design	Prototype implementation	Blockchain / MultiChain	Japan, USA
Mamo N, Martin GM, Desira M, Ellul B, Ebejer JP	2020	European Journal of Human Genetics	Article	Dwarna: a blockchain solution for dynamic consent in biobanking	Sciences (agricultural, biological, chemical, …)	Design	Prototype implementation	Blockchain / Hyperledger Fabric	Austria, Malta
Mathur G, Pandey A, Goyal S	2020	2nd International Conference on Data, Engineering and Applications (IDEA)	Conference Paper	Immutable DNA Sequence Data Transmission for Next Generation Bioinformatics Using Blockchain Technology	Information and Computing Science	Design	System concept	Blockchain / -	India
Neto MM, Marinho CSDS, Coutinho EF, et al.	2020	Proceedings - 2020 IEEE International Conference on Software Architecture	Conference Paper	Research Opportunities for E-health Applications with DNA Sequence Data using Blockchain Technology	Information and Computing Science	Design	Prototype implementation	Blockchain / BigchainDB	Brazil

Authors	Year	Outlet	Document Type	Title	Scientific Field	Approach	Method	DLT Concept / DLT Design	Authors' Countries
		Companion, ICSA-C 2020							
Ozdayi MS, Kantarcioglu M, Malin B	2020	BMC Medical Genomics	Article	Leveraging blockchain for immutable logging and querying across multiple sites	Sciences (agricultural, biological, chemical, …)	Design	Prototype implementation	Blockchain / MultiChain	USA
Pattengale ND, Hudson CM	2020	BMC Medical Genomics	Article	Decentralized genomics audit logging via permissioned blockchain ledgering	Sciences (agricultural, biological, chemical, …)	Design	Prototype implementation	Blockchain / MultiChain	USA
Sami Ullah H, Aslam S, Arjomand N	2020	arXiv	Preprint	Blockchain in Healthcare and Medicine: A Contemporary Research of Applications, Challenges, and Future Perspectives	Information and Computing Science	Review	Narrative review	Blockchain / -	Pakistan, USA
Shuaib K, Saleous H, Zaki N, et al.	2020	2020 IEEE International Conference on Smart Computing (SMARTCOMP)	Conference Paper	A Layered Blockchain Framework for Healthcare and Genomics	Information and Computing Science	Design	Prototype implementation	Blockchain / Consortium Blockchain	United Arab Emirates
Stell A, Chauhan V, Sinnott R	2020	HEALTHINF 2020 - 13th International Conference on Health Informatics, Proceedings; Part of 13th International Joint Conference on Biomedical Engineering Systems and Technologies, BIOSTEC 2020	Conference Paper	Secure audit in support of an adrenal cancer registry	Information and Computing Science	Design	Prototype implementation	Blockchain / Custom Blockchain	Australia
Thiebes S, Schlesner M, Brors B, et al.	2020	European Journal of Human Genetics	Article	Distributed Ledger Technology in genomics: a call for Europe	Sciences (agricultural, biological, chemical, …)	Conceptual	None/unknown	DLT	Germany

Authors	Year	Outlet	Document Type	Title	Scientific Field	Approach	Method	DLT Concept / DLT Design	Authors' Countries
Thiebes S, Kannengießer N, Schmidt-Kraepelin M, et al.	2020	Proceedings of the 52nd Hawaii International Conference on System Sciences	Conference Paper	Beyond data markets: Opportunities and challenges for distributed ledger technology in genomics	Information and Computing Science	Qualitative	Delphi study	DLT	Germany
Uribe D, Waters G	2020	The Journal of The British Blockchain Association	Perspective/Opinions/Views	Privacy Laws, Genomic Data and Non-Fungible Tokens	Information and Computing Science	Conceptual	None/unknown	Blockchain / Ethereum	USA
Warnat-Herresthal S, Schultze H, Shastry KL, et al.	2020	bioRxiv	Preprint	Swarm Learning as a privacy-preserving machine learning approach for disease classification	Sciences (agricultural, biological, chemical, …)	Design	Prototype implementation	Blockchain / -	Germany, Greece, Netherlands
Ya Aung ST, Pluempitiwiriyawej C	2020	InCIT 2020 - 5th International Conference on Information Technology	Conference Paper	Blockchain-Based Implementation for Integration of DNA Profiles Information Systems	Information and Computing Science	Design	Prototype implementation	Blockchain / Ethereum	Thailand
Zhang S, Kim A, Liu D, et al.	2020	arXiv	Preprint	Genie: A Secure, Transparent Sharing and Services Platform for Genetic and Health Data	Information and Computing Science	Design	Prototype implementation	Blockchain / Ethereum	USA
Zmudzin L, Sawicki B	2020	Proceedings of 2020 IEEE 21st International Conference on Computational Problems of Electrical Engineering, CPEE 2020	Conference Paper	Design of Truly Distributed Storage for Large Medical Datasets	Information and Computing Science	Design	System concept	Blockchain / -	Poland
Chattu VK, Sunil TS, Santaji S, et al.	2021	Sleep and Vigilance	Article	Precision Medicine Meets Sleep Medicine: How Can Sleep Health Aid to Reduce the Preventable Burden of Non-communicable Diseases?	Biomedical and Clinical Sciences	Review	Narrative review	Blockchain / -	Canada, India, Iran, Kazakhstan, Malta,

Authors	Year	Outlet	Document Type	Title	Scientific Field	Approach	Method	DLT Concept / DLT Design	Authors' Countries
									USA
Guo X, Khalid MA, Domingos I, et al.	2021	Nature Electronics	Article	Smartphone-based DNA diagnostics for malaria detection using deep learning for local decision support and blockchain technology for security	Engineering	Design	Prototype implementation	Blockchain / Hyperledger Fabric	Uganda, United Kingdom
Pachaury R, Lakshmi CV	2021	Lecture Notes in Mechanical Engineering	Conference Paper	Securing Genomics Data Using Blockchain Technology	Engineering	Design	Prototype implementation	Blockchain / Hyperledger Fabric	India
Racine V	2021	Science and Engineering Ethics	Article	Can Blockchain Solve the Dilemma in the Ethics of Genomic Biobanks?	Arts, Humanities, and Social Sciences	Conceptual	None/unknown	Blockchain / -	USA
Velmovitsky PE, Bublitz FM, Fadrique LX, et al.	2021	JMIR Medical Informatics	Article	Blockchain applications in health care and public health: Increased transparency	Information and Computing Science	Review	Narrative review	Blockchain / -	Canada, Brazil
Warnat-Herresthal S, Schultze H, Shastry KL, et al.	2021	Nature	Article	Swarm Learning for decentralized and confidential clinical machine learning	Sciences (agricultural, biological, chemical, …)	Design	Prototype implementation	Blockchain / Consortium Blockchain	Germany, Greece, Netherlands, USA
