# Supplementary material for: A scoping review of distributed ledger technology in genomics: thematic analysis and directions for future research
Source: J Am Med Inform Assoc. 2022 May 20;29(8):1433–44. doi: 10.1093/jamia/ocac077 (PMC9277639; doi:10.1093/jamia/ocac077)
Supplement: ocac077_supplementary_data [file ocac077_supplementary_data.zip › S5_Summary_of_coding_scheme.pdf]

Table S5. Summary of employed coding scheme

Category	Selected criteria
Publication year	The year in which an examined publication was published.
Publication type	The type of an examined publication.<br>• *Conference paper*: The publication was published as a conference paper or as part of conference proceedings.<br>• *Journal article*: The publication was published as a research article in a scientific journal.<br>• *Perspective*: The publication was published as a perspective-type publication in a scientific journal.<br>• *Preprint*: The publication was published as a preprint on a preprint server (i.e., arXiv, bioRxiv) or elsewhere.
Outlet	The outlet (e.g., journal, conference proceedings, preprint server) in which an examined publication was published.
Scientific discipline	The scientific discipline to which the outlet[a] belongs in which an examined publication was published, oriented toward [1].<br>• *Arts, Humanities and Social Sciences*<br>• *Biomedical and Clinical Sciences*<br>• *Engineering*<br>• *Information and Computing Science*<br>• *Sciences (agricultural, biological, chemical, earth, environmental, mathematical, physical)*
Research approach	The general research approach of an examined publication.<br>• *Conceptual*: The publication is of conceptual nature (i.e., the concept of interest is investigated on an abstract level).<br>• *Design*: The publication is concerned with the development of a prototype or system concept.<br>• *Mixed methods*: The examined publication employs multiple, different types of research approaches (e.g., qualitative and quantitative).<br>• *Qualitative*: The research approach of the examined publication is of qualitative nature.<br>• *Quantitative*: The research approach of the examined publication is of quantitative nature.<br>• *Review*: The examined publication is a literature review.
Research method	The concrete research method employed in an examined publication.<br>• *Case study*<br>• *Delphi study*<br>• *Narrative review*<br>• *None/unknown*<br>• *Prototype implementation*<br>• *Prototype + focus group*<br>• *Soft systems method*<br>• *System concept*
DLT concept	The DLT concept investigated by an examined publication according to [2].<br>• *Not specified*<br>• *Blockchain*

Category	Selected criteria
DLT design	The DLT design investigated by an examined publication according to [2].<br>• *Not specified*<br>• *BigchainDB*<br>• *Consortium Blockchain*<br>• *Corda*<br>• *Custom Blockchain*<br>• *Custom partial Blockchain*<br>• *Ethereum*<br>• *Exonum*<br>• *Hyperledger Fabric*<br>• *Hyperledger Indy*<br>• *MultiChain*

[a]To determine an outlet's scientific discipline, we used its category in the Clarivate Analytics Journal Citation Reports. If this information was not available, we used its Scopus categorization instead. If this information was also not available, a best fitting discipline was assigned based on a discussion among all authors. For example, preprints in bioRxiv are classified as *"Information and Computing Science"* because the platform is focused on biological work. Likewise, if multiple categories were available in Clarivate Analytics Journal Citation Reports or Scopus a best fitting discipline was assigned based on a discussion among all authors. For example, articles in the Journal of Medical Internet Research are classified as *"Information and Computing Science"* despite the journal's category also allowing for a classification as *"Biomedical and Clinical Sciences".*

REFERENCES

1. Statistics ABo. Australian and New Zealand Standard Research Classification (Anzsrc). Secondary Australian and New Zealand Standard Research Classification (Anzsrc) 2020. https://www.abs.gov.au/statistics/classifications/australian-and-new-zealand-standard-research-classification-anzsrc/latest-release.
2. Kannengießer N, Lins S, Dehling T, Sunyaev A. Trade-Offs between Distributed Ledger Technology Characteristics. ACM Computing Surveys 2020;**53**(2):1-37 doi: 10.1145/3379463.
