# Supplementary material for: A scoping review of distributed ledger technology in genomics: thematic analysis and directions for future research
Source: J Am Med Inform Assoc. 2022 May 20;29(8):1433–44. doi: 10.1093/jamia/ocac077 (PMC9277639; doi:10.1093/jamia/ocac077)
Supplement: ocac077_supplementary_data [file ocac077_supplementary_data.zip › S7_Table_of_themes_per_article.pdf]

Table S7. List of themes discussed per article

Author	Data economy and sharing	Data management	Data protection	Data storage	Decentralized data analysis	Proof of Useful Work	ELSI
Ileri AM, et al. [1]		X	X	X		X	X
Engelhardt M [2]	X	X	X				X
Chavali LN, et al. [3]	X	X	X	X			X
Dambrot SM [4]			X	X	X		
Gökalp E, et al. [5]	X		X	X			X
Grishin D, et al. [6]	X	X	X	X	X		X
Langley PC, et al. [7]	X	X					X
Lee S-J, et al. [8]	X	X	X	X			
Ozercan HI, et al. [9]	X	X		X	X		X
Zhang X, et al. [10]	X	X	X	X			X
Ahmed E, et al. [11]	X	X					X
Akshayaa S, et al. [12]	X		X				
Carlini R, et al. [13]	X	X	X				X
Dimitrov DV [14]	X	X	X	X			
Iyer V, et al. [15]	X	X	X		X		X
Jin X-L, et al. [16]	X	X	X	X			X
Justinia T[17]	X	X	X	X			X
Kuo T-T, et al. [18]					X		X
Mackey TK, et al. [19]	X	X	X	X			X
Preethi V, et al. [20]	X	X	X	X	X		
Shabani M [21]	X	X	X	X			X
Sharma R, et al. [22]	X	X	X	X			X
Venner E, et al. [23]	X	X	X	X			X
Zhang Y, et al. [24]	X	X	X	X	X	X	X
Zhavoronkov A, et al. [25]	X						X
Zimmerman N, et al. [26]	X	X	X	X	X		
Aung STY, et al. [27]	X	X	X	X			X
Carlini F, et al. [28]	X	X	X	X	X		X
Chernomoretz A, et al. [29]			X				
Evangelatos N, et al. [30]	X	X	X				X
Glicksberg BS, et al. [31]	X	X	X	X			X
Gürsoy G, et al. [32]		X	X	X			X
Gürsoy G, et al. [33]		X	X	X			X
Gürsoy G, et al. [34]	X	X	X	X	X		
Hendricks-Sturrup RM, et al. [35]	X		X				X
Jung T, et al. [36]	X	X	X	X	X	X	X
Kuo T-T, et al. [37]	X	X	X		X		
Kuo T-T [38]	X		X	X			X
Lemieux VL [39]	X	X	X	X	X		X
Ma S, et al. [40]		X					
Mamo N, et al. [41]	X	X	X	X	X		X
Mathur G, et al. [42]	X		X	X			
Neto MM, et al. [43]	X	X	X	X	X		X
Ozdayi MS, et al. [44]		X		X			
Pattengale ND, et al. [45]	X	X	X	X			X

Author	Data economy and sharing	Data management	Data protection	Data storage	Decentralized data analysis	Proof of Useful Work	ELSI
Shuaib K, et al. [46]	X	X	X	X			X
Stell A, et al. [47]		X	X				X
Thiebes S, et al. [48]	X	X	X	X			X
Thiebes S, et al. [49]	X	X	X	X		X	X
Ullah HS, et al. [50]	X	X	X	X			X
Uribe D, et al. [51]	X	X		X	X		X
Warnat-Herresthal S, et al. [52]	X		X		X		X
Zhang S, et al. [53]	X	X	X	X			X
Zmudzin L, et al. [54]	X	X	X	X			X
Chattu VK, et al. [55]	X	X	X	X			X
Guo X, et al. [56]	X	X	X	X	X		X
Pachaury R, et al. [57]	X	X	X	X			X
Racine V [58]	X	X	X	X			X
Velmovitsky PE, et al. [59]	X	X	X	X			X
Warnat-Herresthal S, et al. [60]	X	X	X	X	X		

REFERENCES

1. Ileri AM, Ozercan HI, Gundogdu A, Senol AK, Yusuf Ozkaya M, Alkan C. Coinami: A Cryptocurrency with DNA Sequence Alignment as Proof-of-Work. https://arxiv.org/abs/1602.03031: arXiv, 2016:arXiv:1602.03031.
2. Engelhardt MA. Hitching Healthcare to the Chain: An Introduction to Blockchain Technology in the Healthcare Sector. Technology Innovation Management Review 2017;**7**(10).
3. Chavali LN, Prashanti NL, Sujatha K, Rajasheker G, Kavi Kishor PB. The Emergence of Blockchain Technology and Its Impact in Biotechnology, Pharmacy and Life Sciences. Current Trends in Biotechnology and Pharmacy 2018;**12**(3):304-10.
4. Dambrot SM. Regene: Blockchain Backup of Genome Data and Restoration of Pre-Engineered Expressed Phenotype. 2018 9th IEEE Annual Ubiquitous Computing, Electronics and Mobile Communication Conference, UEMCON 2018, 2018:945-50.
5. Gökalp E, Gökalp MO, Çoban S, Eren PE. Analysing Opportunities and Challenges of Integrated Blockchain Technologies in Healthcare. Information Systems: Research, Development, Applications, Education, 2018:174-83.
6. Grishin D, Obbad K, Estep P, et al. Accelerating Genomic Data Generation and Facilitating Genomic Data Access Using Decentralization, Privacy-Preserving Technologies and Equitable Compensation. Blockchain in Healthcare Today 2018;**1**:1-23 doi: 10.30953/bhty.v1.34.
7. Langley PC, Martin RE. If You Build It Will They Come? Patients, Providers and Blockchains in Health Technology Assessment. Innov Pharm 2018;**9**(4) doi: 10.24926/iip.v9i4.1453.
8. Lee S-J, Cho G-Y, Ikeno F, Lee T-R. Baqalc: Blockchain Applied Lossless Efficient Transmission of DNA Sequencing Data for Next Generation Medical Informatics. Applied Sciences 2018;**8**(9) doi: 10.3390/app8091471.
9. Ozercan HI, Ileri AM, Ayday E, Alkan C. Realizing the Potential of Blockchain Technologies in Genomics. Genome Res 2018;**28**(9):1255-63 doi: 10.1101/gr.207464.116.
10. Zhang X, Sharma R, Wingreen S. Block-Chaining in Precision Healthcare: A Design Research Approach. Proceedings of the 22nd Pacific Asia Conference on Information

Systems - Opportunities and Challenges for the Digitized Society: Are We Ready?, PACIS 2018, 2018.
11. Ahmed E, Shabani M. DNA Data Marketplace: An Analysis of the Ethical Concerns Regarding the Participation of the Individuals. Front Genet 2019;**10**:1107 doi: 10.3389/fgene.2019.01107.
12. Akshayaa S, Vidhya R, Hima vyshnavi AM, Krishnan Namboori PK. Exploring Pain Insensitivity Inducing Gene Zfhx2 by Using Deep Convolutional Neural Network. 2019 3rd International Conference on Computing Methodologies and Communication (ICCMC), 2019:68-72.
13. Carlini R, Carlini F, Dalla Palma S, Pareschi R. Genesy: A Blockchain-Based Platform for DNA Sequencing. CEUR Workshop Proceedings, 2019:68-72.
14. Dimitrov DV. Blockchain Applications for Healthcare Data Management. Healthc Inform Res 2019;**25**(1):51-56 doi: 10.4258/hir.2019.25.1.51.
15. Iyer V, Vyshnavi AMH, Iyer S, Namboori PKK. An Ai Driven Genomic Profiling System and Secure Data Sharing Using Dlt for Cancer Patients. 2019 IEEE Bombay Section Signature Conference (IBSSC), 2019:1-5.
16. Jin XL, Zhang M, Zhou Z, Yu X. Application of a Blockchain Platform to Manage and Secure Personal Genomic Data: A Case Study of Lifecode.Ai in China. J Med Internet Res 2019;**21**(9):e13587 doi: 10.2196/13587.
17. Justinia T. Blockchain Technologies: Opportunities for Solving Real-World Problems in Healthcare and Biomedical Sciences. Acta Inform Med 2019;**27**(4):284-91 doi: 10.5455/aim.2019.27.284-291.
18. Kuo TT, Gabriel RA, Ohno-Machado L. Fair Compute Loads Enabled by Blockchain: Sharing Models by Alternating Client and Server Roles. J Am Med Inform Assoc 2019;**26**(5):392-403 doi: 10.1093/jamia/ocy180.
19. Mackey TK, Kuo TT, Gummadi B, et al. 'Fit-for-Purpose?' - Challenges and Opportunities for Applications of Blockchain Technology in the Future of Healthcare. BMC Med 2019;**17**(1):68 doi: 10.1186/s12916-019-1296-7.
20. Preethi V, Surve S. Blockchain Enabled DNA Banking and Comparative Analysis Using Hyperledger Fabric. International Journal of Engineering and Advanced Technology 2019;**8**(4):4.
21. Shabani M. Blockchain-Based Platforms for Genomic Data Sharing: A De-Centralized Approach in Response to the Governance Problems? J Am Med Inform Assoc 2019;**26**(1):76-80 doi: 10.1093/jamia/ocy149.
22. Sharma R, Zhang C, Wingreen SC, Kshetri N, Zahid A. Design of Blockchain-Based Precision Health-Care Using Soft Systems Methodology. Industrial Management & Data Systems 2019;**120**(3):608-32 doi: 10.1108/imds-07-2019-0401.
23. Venner E, Murugan M, Hale W, et al. Arbor: An Identity and Security Solution for Clinical Reporting. J Am Med Inform Assoc 2019;**26**(11):1370-74 doi: 10.1093/jamia/ocz107.
24. Zhang Y, Zhao X, Li X, Zhong M, Curtis C, Chen C. Enabling Privacy-Preserving Sharing of Genomic Data for Gwass in Decentralized Networks. Proceedings of the Twelfth ACM International Conference on Web Search and Data Mining, 2019:204-12.
25. Zhavoronkov A, Church G. The Advent of Human Life Data Economics. Trends Mol Med. 2019;**25**(6):566-70 doi: 10.1016/j.molmed.2019.05.002.
26. Zimmermann N, Tatonetti NP, Dudley JT. A Marketplace for Health: Opportunities and Challenges for Biomedical Blockchains. http://tatonettilab.org/resources/Blockchain_Perspective_ZTD.pdf, 2019:7.
27. Blockchain-Based Implementation for Integration of DNA Profiles Information Systems. InCIT 2020 - 5th International Conference on Information Technology; 2020.
28. Carlini F, Carlini R, Palma SD, Pareschi R, Zappone F, Albanese D. The Genesy Model for a Blockchain-Based Fair Ecosystem of Genomic Data. 2020 Seventh International Conference on Software Defined Systems (SDS), 2020:183-89.
29. Chernomoretz A, Balparda M, La Grutta L, et al. Genis, an Open-Source Multi-Tier Forensic DNA Information System. Forensic Science International: Reports 2020;**2** doi: 10.1016/j.fsir.2020.100132.

30. Evangelatos N, Upadya SP, Venne J, et al. Digital Transformation and Governance Innovation for Public Biobanks and Free/Libre Open Source Software Using a Blockchain Technology. OMICS 2020;**24**(5):278-85 doi: 10.1089/omi.2019.0178.
31. Glicksberg BS, Burns S, Currie R, et al. Blockchain-Authenticated Sharing of Genomic and Clinical Outcomes Data of Patients with Cancer: A Prospective Cohort Study. J Med Internet Res 2020;**22**(3):e16810 doi: 10.2196/16810.
32. Gürsoy G, Bjornson R, Green ME, Gerstein M. Using Blockchain to Log Genome Dataset Access: Efficient Storage and Query. BMC Med Genomics 2020;**13**(Suppl 7):78 doi: 10.1186/s12920-020-0716-z.
33. Gürsoy G, Brannon CM, Gerstein M. Using Ethereum Blockchain to Store and Query Pharmacogenomics Data Via Smart Contracts. BMC Med Genomics 2020;**13**(1):74 doi: 10.1186/s12920-020-00732-x.
34. Gürsoy G, Brannon CM, Wagner S, Gerstein M. Storing and Analyzing a Genome on a Blockchain. https://www.biorxiv.org/content/10.1101/2020.03.03.975334v2: bioRxiv, 2020.
35. Hendricks-Sturrup RM, Lu CY. What Motivates the Sharing of Consumer-Generated Genomic Information? SAGE Open Med 2020;**8**:2050312120915400 doi: 10.1177/2050312120915400.
36. Jung T, Leu R. Blockchain's Potential to Address Issues in Genomics Research and How It Is Being Used Today. University of Western Ontario Medical Journal 2020;**88**(S):13-15 doi: 10.5206/uwomj.v88iS.8233.
37. Kuo TT, Gabriel RA, Cidambi KR, Ohno-Machado L. Expectation Propagation Logistic Regression on Permissioned Blockchain (Explorerchain): Decentralized Online Healthcare/Genomics Predictive Model Learning. J Am Med Inform Assoc 2020;**27**(5):747-56 doi: 10.1093/jamia/ocaa023.
38. Kuo TT. The Anatomy of a Distributed Predictive Modeling Framework: Online Learning, Blockchain Network, and Consensus Algorithm. JAMIA Open 2020;**3**(2):201-08 doi: 10.1093/jamiaopen/ooaa017.
39. Lemieux VL, Hofman D, Hamouda H, et al. Having Our "Omic" Cake and Eating It Too?: Evaluating User Response to Using Blockchain Technology for Private and Secure Health Data Management and Sharing. Frontiers in Blockchain 2021;**3**:59.
40. Ma S, Cao Y, Xiong L. Efficient Logging and Querying for Blockchain-Based Cross-Site Genomic Dataset Access Audit. BMC Med Genomics 2020;**13**(Suppl 7):91 doi: 10.1186/s12920-020-0725-y.
41. Mamo N, Martin GM, Desira M, Ellul B, Ebejer JP. Dwarna: A Blockchain Solution for Dynamic Consent in Biobanking. Eur J Hum Genet 2020;**28**(5):609-26 doi: 10.1038/s41431-019-0560-9.
42. Mathur G, Pandey A, Goyal S. Immutable DNA Sequence Data Transmission for Next Generation Bioinformatics Using Blockchain Technology. 2nd International Conference on Data, Engineering and Applications (IDEA), 2020:1-6.
43. Neto MM, S.Marinho CSd, Coutinho EF, Moreira LO, Machado JdC, Souza JNd. Research Opportunities for E-Health Applications with DNA Sequence Data Using Blockchain Technology. 2020 IEEE International Conference on Software Architecture Companion (ICSA-C), 2020:95-102.
44. Ozdayi MS, Kantarcioglu M, Malin B. Leveraging Blockchain for Immutable Logging and Querying across Multiple Sites. BMC Med Genomics 2020;**13**(Suppl 7):82 doi: 10.1186/s12920-020-0721-2.
45. Pattengale ND, Hudson CM. Decentralized Genomics Audit Logging Via Permissioned Blockchain Ledgering. BMC Med Genomics 2020;**13**(Suppl 7):102 doi: 10.1186/s12920-020-0720-3.
46. Shuaib K, Saleous H, Zaki N, Dankar F. A Layered Blockchain Framework for Healthcare and Genomics. 2020 IEEE International Conference on Smart Computing (SMARTCOMP), 2020:156-63.
47. Stell A, Chauhan V, Sinnott R. Secure Audit in Support of an Adrenal Cancer Registry. Proceedings of the 13th International Joint Conference on Biomedical Engineering Systems and Technologies, 2020:237-48.

48. Thiebes S, Schlesner M, Brors B, Sunyaev A. Distributed Ledger Technology in Genomics: A Call for Europe. Eur J Hum Genet 2020;**28**(2):139-40 doi: 10.1038/s41431-019-0512-4.
49. Thiebes S, Kannengießer N, Schmidt-Kraepelin M, Sunyaev A. Beyond Data Markets: Opportunities and Challenges for Distributed Ledger Technology in Genomics. Proceedings of the 52nd Hawaii International Conference on System Sciences, 2020:10.
50. Ullah HS, Alsam S, Arjomand N. Blockchain in Healthcare and Medicine: A Contemporary Research of Applications, Challenges, and Future Perspectives. https://arxiv.org/abs/2004.06795, 2020:16.
51. Uribe D, Waters G. Privacy Laws, Non-Fungible Tokens, and Genomics. The Journal of The British Blockchain Association 2020;**3**(2):1-10 doi: 10.31585/jbba-3-2-(5)2020.
52. Warnat-Herresthal S, Schultze H, Shastry KL, et al. Swarm Learning as a Privacy-Preserving Machine Learning Approach for Disease Classification. https://www.biorxiv.org/content/10.1101/2020.06.25.171009v2: bioRxiv, 2020.
53. Zhang S, Kim A, Liu D, et al. Genie: A Secure, Transparent Sharing and Services Platform for Genetic and Health Data. https://arxiv.org/abs/1811.01431, 2020:10.
54. Design of Truly Distributed Storage for Large Medical Datasets. Proceedings of 2020 IEEE 21st International Conference on Computational Problems of Electrical Engineering, CPEE 2020; 2020.
55. Chattu VK, Sunil TS, Santaji S, et al. Precision Medicine Meets Sleep Medicine: How Can Sleep Health Aid to Reduce the Preventable Burden of Non-Communicable Diseases? Sleep and Vigilance 2021;**5**(2):179-88 doi: 10.1007/s41782-021-00149-1.
56. Guo X, Khalid MA, Domingos I, et al. Smartphone-Based DNA Diagnostics for Malaria Detection Using Deep Learning for Local Decision Support and Blockchain Technology for Security. Nature Electronics 2021;**4**(8):615-24 doi: 10.1038/s41928-021-00612-x.
57. Pachaury R, Lakshmi CV. Securing Genomics Data Using Blockchain Technology. Advances in Systems Engineering, 2021:473-80.
58. Racine V. Can Blockchain Solve the Dilemma in the Ethics of Genomic Biobanks? Sci Eng Ethics 2021;**27**(3):35 doi: 10.1007/s11948-021-00311-y.
59. Velmovitsky PE, Bublitz FM, Fadrique LX, Morita PP. Blockchain Applications in Health Care and Public Health: Increased Transparency. JMIR Med Inform 2021;**9**(6):e20713 doi: 10.2196/20713.
60. Warnat-Herresthal S, Schultze H, Shastry KL, et al. Swarm Learning for Decentralized and Confidential Clinical Machine Learning. Nature 2021;**594**(7862):265-70 doi: 10.1038/s41586-021-03583-3.
