# Supplementary material for: A scoping review of distributed ledger technology in genomics: thematic analysis and directions for future research
Source: J Am Med Inform Assoc. 2022 May 20;29(8):1433–44. doi: 10.1093/jamia/ocac077 (PMC9277639; doi:10.1093/jamia/ocac077)
Supplement: ocac077_supplementary_data [file ocac077_supplementary_data.zip › S8_Exemplary_Research_Questions.pdf]

Table S8. Exemplary research questions for each presented research direction

Research direction	Exemplary research questions
1. Explore opportunities for the application of DLT concepts other than Blockchain	• RQ1.1: What DLT concepts other than Blockchain are suitable for the genomics context?<br>• RQ1.2: How do the different properties of DLT concepts relate to the requirements of applications scenarios in genomics?<br>• RQ1.3: How can current limitations of DLT-based systems in genomics be addressed using other DLT concepts (i.e., other than Blockchain)?
2. Investigate people's attitudes and behaviors regarding the commodification of genetic data through DLT-based genetic data markets	• RQ2.1: What are individuals' (e.g., patients', professionals') attitudes toward DLT-based data markets for genetic data?<br>• RQ2.2: What are drivers or inhibitors for the adoption of DLT-based data markets for genetic data for different types of users (e.g., data donors, data consumers).<br>• RQ2.3: How do DLT-based data markets foster the commodification of genetic data and what are the implications of this?
3. Examine opportunities for joint consent management via DLT	• RQ3.1: How can DLT enable the management of joint consent (e.g., for family members) regarding the disclosure of genetic data?<br>• RQ3.2: Which joint consent schemes are applicable in the context of genetic data and genomic research?<br>• RQ3.3: How to design DLT-based joint consent management systems?
4. Investigate and evaluate data storage models appropriate for DLT	• RQ4.1: What types of data (in the realms of genetic data) are suitable for on-ledger storage and what data are suitable for off-ledger storage?<br>• RQ4.2: How to efficiently store large amounts of genetic data on-ledger?<br>• RQ4.3: How to design DLT-based systems in genomics with combined on-ledger and off-ledger data storage?
5. Research the regulation-compliant use of DLT in healthcare information systems	• RQ5.1: How do current privacy regulations (e.g., HIPAA, GDPR) impact (positively or negatively) the application of DLT in genomics?<br>• RQ5.2: What are regulatory hurdles with respect to the application of DLT in genomics?<br>• RQ5.3: How to design DLT-based systems for genomics in accordance with contemporary regulations?<br>• RQ5.4: How might future regulations impact the application of DLT in genomics?
6. Investigate alternative consensus mechanisms based on PoUW	• RQ6.1: What are the advantages or disadvantages of PoUW-based consensus mechanisms over other consensus mechanisms in the context of genomics?<br>• RQ6.2: How can PoUW be effectively utilized for DLT-based systems in genomics?<br>• RQ6.3: Which genomic analysis tasks are suitable for PoUW in DLT-based systems

Research direction	Exemplary research questions
	• RQ6.4: How to decrease associated centralization issues for systems in genomics based on PoUW work?
7. Explore DLT-enabled approaches for the protection of genetic data ensuring user privacy	• RQ7.1: What are the genomic privacy implications when sharing data via extant DLT systems?<br>• RQ7.2: How can DLT be used to ensure genomic privacy when sharing data?<br>• RQ7.3: How can pseudonymization of genetic data ensure genomic privacy in DLT-based systems?<br>• RQ7.4: How do DLT-specific systemic risks (e.g., through key management) affect users' interaction with DLT-based systems managing genetic data?
**Glossary:**<br>DLT = Distributed Ledger Technology<br>PoUW = Proof of Useful Work	
