# Supplementary figures and images for: A scoping review of distributed ledger technology in genomics: thematic analysis and directions for future research
Source: J Am Med Inform Assoc. 2022 May 20;29(8):1433–44. doi: 10.1093/jamia/ocac077 (PMC9277639; doi:10.1093/jamia/ocac077)

Figure S6. Mind map of identified focal research themes (yellow), subthemes (grey), and aspects (white).

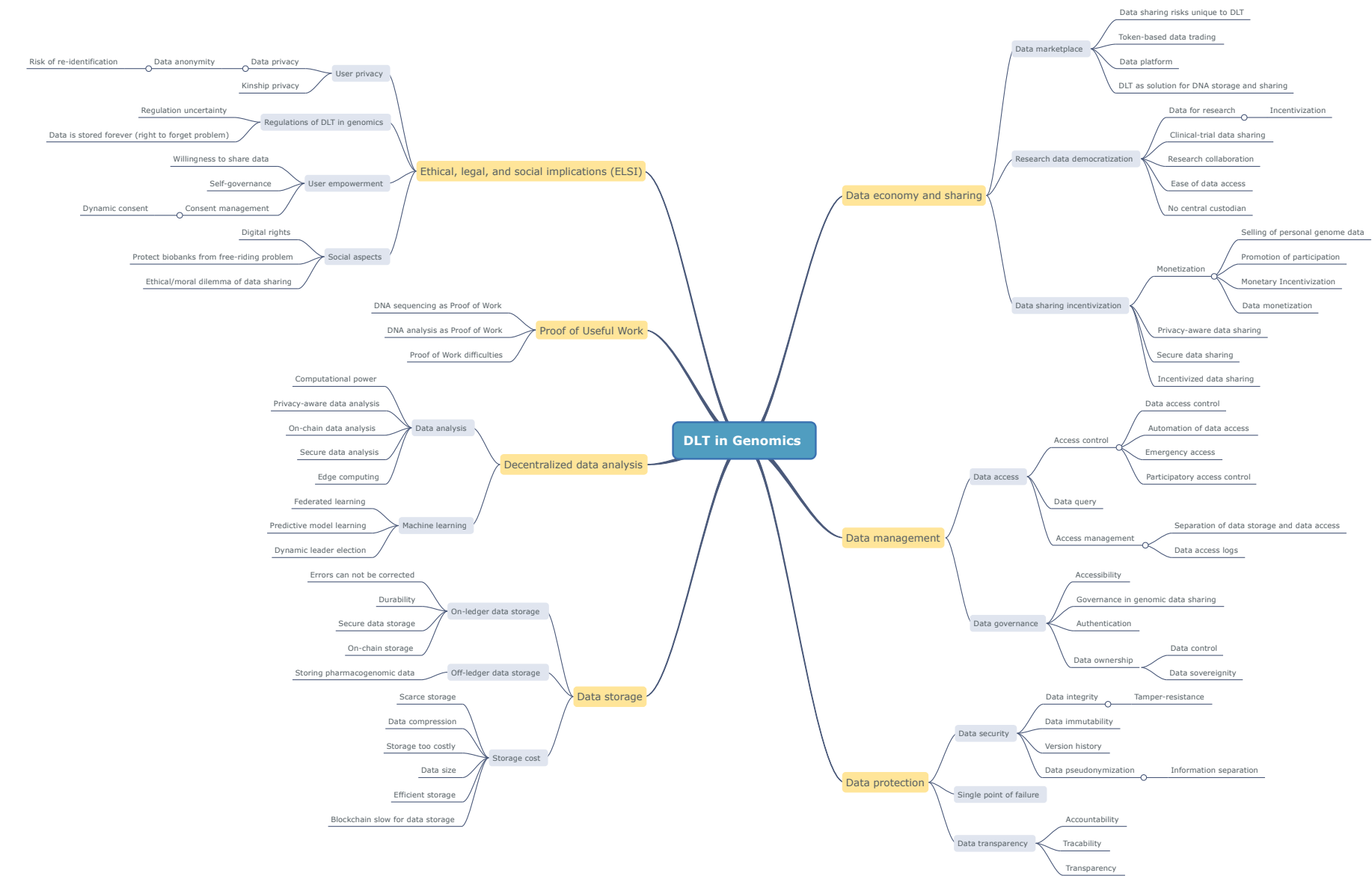

Supplement: ocac077_supplementary_data [file ocac077_supplementary_data.zip › S6_Mindmap_of_research_themes.pdf]
